# Activation of Serotonin 2C Receptors in Dopamine Neurons Inhibits Binge-like Eating in Mice

**DOI:** 10.1016/j.biopsych.2016.06.005

**Published:** 2017-05-01

**Authors:** Pingwen Xu, Yanlin He, Xuehong Cao, Lourdes Valencia-Torres, Xiaofeng Yan, Kenji Saito, Chunmei Wang, Yongjie Yang, Antentor Hinton, Liangru Zhu, Gang Shu, Martin G. Myers, Qi Wu, Qingchun Tong, Lora K. Heisler, Yong Xu

**Affiliations:** a Department of Pediatrics, Baylor College of Medicine, Houston, Texas; b Children’s Nutrition Research Center, and Department of Molecular and Cellular Biology, Baylor College of Medicine, Houston, Texas; c Rowett Institute of Nutrition and Health, Foresterhill, Aberdeen, United Kingdom; d Department of Gastroenterology, Union Hospital, Tongji Medical College, Huazhong University of Sciences and Technology, Wuhan, Hubei, China; e Department of Internal Medicine and Molecular and Integrative Physiology, University of Michigan, Ann Arbor, Michigan; f Brown Foundation Institute of Molecular Medicine, University of Texas Health Science Center at Houston, Houston, Texas

**Keywords:** Binge eating, Dopamine, Lorcaserin, Neuron, Receptor, Serotonin

## Abstract

**Background:**

Neural networks that regulate binge eating remain to be identified, and effective treatments for binge eating are limited.

**Methods:**

We combined neuroanatomic, pharmacologic, electrophysiological, Cre-lox, and chemogenetic approaches to investigate the functions of 5-hydroxytryptamine (5-HT) 2C receptor (5-HT_2C_R) expressed by dopamine (DA) neurons in the regulation of binge-like eating behavior in mice.

**Results:**

We showed that 5-HT stimulates DA neural activity through a 5-HT_2C_R-mediated mechanism, and activation of this midbrain 5-HT→DA neural circuit effectively inhibits binge-like eating behavior in mice. Notably, 5-HT medications, including fluoxetine, d-fenfluramine, and lorcaserin (a selective 5-HT_2C_R agonist), act on 5-HT_2C_Rs expressed by DA neurons to inhibit binge-like eating in mice.

**Conclusions:**

We identified the 5-HT_2C_R population in DA neurons as one potential target for antibinge therapies, and provided preclinical evidence that 5-HT_2C_R agonists could be used to treat binge eating.

Binge eating, defined as the ingestion of a large amount of food in short timeframe, is a central feature of eating disorders such as bulimia nervosa and binge eating disorder (1, 2) and is prevalent in approximately 5% of U.S. adults (3). The pathophysiology of binge eating in humans is not well understood, and the effective treatments for this condition are limited.

Impaired brain 5-hydroxytryptamine (5-HT; serotonin) signaling is linked to the development of binge eating in humans (4, 5, 6, 7). For example, binge eating patients are found to have increased brain 5-HT uptake and consequently decreased 5-HT content (4). In addition, effects of 5-HT precursor L-tryptophan are significantly blunted in binge eating patients, presumably due to dysfunctions of 5-HT receptors and/or tryptophan hydroxylase-2 (TPH2) (4), the enzyme that synthesizes 5-HT in the brain. Furthermore, selective serotonin reuptake inhibitors that increase central 5-HT content, such as fluoxetine, suppress binge eating (8, 9, 10, 11, 12, 13). Notably, d-fenfluramine (d-Fen), a drug that increases 5-HT content (14), effectively reduces the number of binge episodes in severely obese patients (15, 16). However, d-Fen was withdrawn from clinical use because of adverse cardiopulmonary events (17). Due to the effect of these 5-HT compounds on binge eating, efforts have been focused on understanding the mechanisms underlying their antibinge benefits, with an attempt to facilitate the development of new pharmaceutical agents that suppress binge eating, with fewer side effects.

The central dopamine (DA) system is also implicated in the pathophysiology of binge eating (18). For instance, human genetic studies have shown an increased frequency of DA transporter (DAT) and D_2_ receptor polymorphisms with binge pathology (19). Rats that binge sucrose demonstrate increased DA release in the nucleus accumbens (20, 21), a ventral striatal structure that receives dopaminergic projections from the ventral tegmental area (VTA) (22, 23). Interestingly, deep brain stimulation targeting the shell of nucleus accumbens potently inhibits binge eating in mice (24). Furthermore, raclopride, a nonselective D_2_ receptor antagonist that presumably activates DA neurons (25), suppresses binge eating in rats (26, 27). Together, these suggest a potential role of DA neurons in the pathophysiology and treatment of binge eating. Interestingly, 5-HT neurons in the dorsal raphe nuclei (DRN) directly project to and synapse on DA neurons in the VTA (28), which provides an anatomic framework for an interaction between 5-HT and DA g., between 5-HT and DA); please supply second thing. -->that may have relevance to the regulation of binge eating.

An intermittent high-fat diet (HFD) exposure paradigm has been used by others and us to induce binge-like eating behavior in mice and rats that in many ways simulates binge eating in humans (24, 29, 30, 31). In the current study, we used this binge-inducing paradigm to first confirm antibinge efficacy of fluoxetine and d-Fen in wild-type (WT) mice. We then combined a variety of genetic mouse models to determine whether serotonin 2C receptors (5-HT_2C_Rs) expressed in DA neurons are required or sufficient to mediate inhibitory effects of these 5-HT drugs on binge-like eating behavior. Furthermore, we assessed whether a Food and Drug Administration–approved 5-HT_2C_R agonist (lorcaserin) acts on this 5-HT_2C_R population to inhibit binge-like eating behavior in mice. Finally, electrophysiological recordings and designer receptors exclusively activated by designer drugs (DREADD) approaches were used to determine whether 5-HT drugs inhibit binge-like eating via regulating DA neural activity.

## Methods and Materials

### Mice

All the transgenetic breeders have been backcrossed to C57BL/6 background for more than 12 generations (see Supplement for detailed breeding strategies). Mice were housed in a temperature-controlled environment in groups of two to five at 22°C to 24°C using a 12-hour light/dark cycle. The mice were fed standard chow (6.5% fat, #2920, Harlan-Teklad, Madison, WI) until training and assessment of binge-like eating behavior. Water was provided ad libitum.

### Effects of 5-HT Compounds on Binge-like Eating Behavior

Mice (12 weeks of age) were subjected to either “intermittent” HFD exposure or “continuous” chow/HFD exposure, as described in the Supplement. On the binge assessment days, mice received intraperitoneal (i.p.) injections of saline, or various 5-HT compounds, at 10:30 am (30 minutes before assessment). The same mice received all different injections in different cycles. The order of injections was randomized to avoid potential sequence effects.

### DREADD-Induced Activation of DA Neurons and Binge-like Eating Behavior

DAT-CreER mice (male and female, 12 weeks of age) were anesthetized with isoflurane and received stereotaxic injections of the excitatory adeno-associated virus (AAV)-hM3Dq-mCherry DREADD virus (200 nL/site; University of North Carolina Gene Therapy Center, Chapel Hill, NC) into both sites of VTA or into both sites of the substantia nigra (SN). These mice also received i.p. injections of tamoxifen (4 mg/mouse). After a 7-day recovery, mice were subjected to the intermittent HFD exposure to induce binge-like eating, as we have described. On the binge assessment days, mice received i.p. injections of saline or clozapine N-oxide ([CNO]; 1 mg/kg #HY-17366; MedChem Express, Monmouth Junction, NJ), at 10:30 am (30 minutes before assessment). To assess binge-like eating, 2.5-hour HFD intake was measured. The same mice received both saline and CNO injections in different cycles. The order of injections was randomized to avoid potential sequence effects.

### Statistics

The minimum sample size was predetermined by the nature of experiments. For most of the physiological readouts (e.g., food intake), at least six mice per group were included. For histology studies, two or three mice were included in each group. For electrophysiological studies, at least 17 neurons in each genotype or condition were included. The data are presented as mean ± SEM. Statistical analyses were performed using GraphPad Prism (GraphPad Software, San Diego, CA) to evaluate normal distribution and variations within and among groups. Methods of statistical analyses were chosen based on the design of each experiment and are indicated in figure legends. *p* < .05 was considered to be statistically significant.

See Supplement for detailed methods.

## Results

### 5-HT_2C_Rs Largely Mediate Effects of Fluoxetine and d-Fen to Inhibit Binge-like Eating in Mice

**Figure 1:**
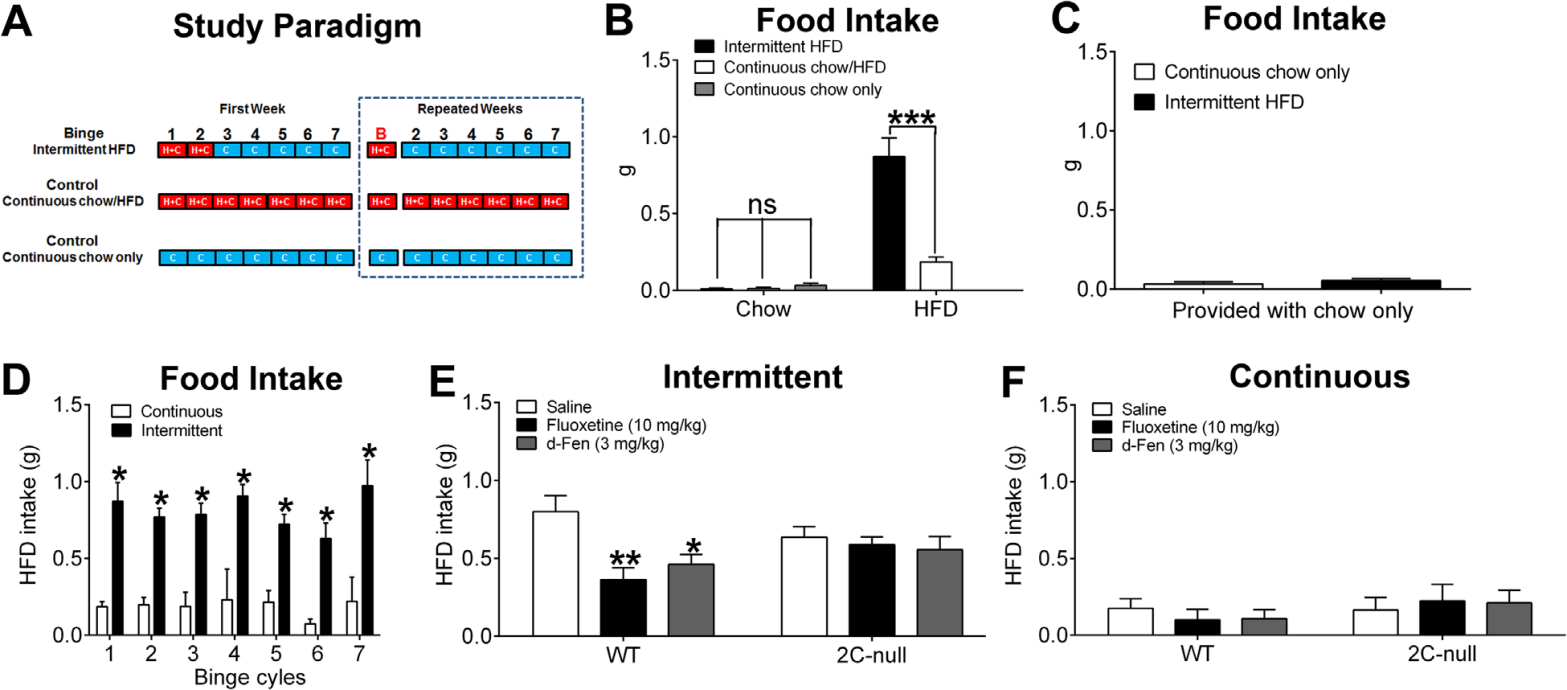
5-Hydroxytryptamine drugs inhibit binge-like eating via 5-hydroxytryptamine 2C receptor–mediated mechanisms. **(A)** Study paradigm to train binge and control mice. In the first week, binge mice were exposed to both a regular chow (C) and high-fat diet (HFD [H]) for 2 days (from Monday 11:00 am to Wednesday 11:00 am), and then exposed to only chow for the rest of the week. On the binge day (red B, Monday of the second week), HFD was returned to cages at 11:00 am and removed at 11:00 am on Tuesday. The same cycles were repeated as the second week. As control subjects, one group of mice was provided with chow and HFD all the time (continuous chow/HFD feeding), and another group was fed with chow (continuous chow only). **(B)** We measured 2.5-hour diet intake in intermittent mice and two control groups from 11:00 am to 1:30 pm on binge day (red B). *n* = 7 or 10 per group. Results are shown as mean ± SEM. ^***^p < .001 in two-way analyses of variance followed by post hoc Sidak tests. **(C)** We measured 2.5-hour chow intake in intermittent mice and continuous chow mice from 11:00 am to 1:30 pm on binge day (red B) when only chow was provided. *n* = 12 or 14 per group. Results are shown as mean ± SEM. **(D)** We measured 2.5-hour HFD intake on the binge day of seven repeated cycles in mice exposed to either continuous chow/HFD or intermittent HFD feeding. *n* = 6 or 7 per group. Results are shown as mean ± SEM. ^*^p < .05 between continuous and intermittent groups at the same cycle in two-way analyses of variance followed by post hoc Sidak tests. **(E)** Effects of intraperitoneal injections of saline, fluoxetine (10 mg/kg), or d-fenfluramine (d-Fen) (3 mg/kg) on binge-like eating (2.5-hour HFD intake) in wild-type (WT) or 2C-null mice exposed to intermittent HFD feeding. *n* = 6 to 10 per group. Results are shown as mean ± SEM. ^*^p < .05 and ^**^p < .01 between drug treatment and saline treatment in the same genotype in two-way analyses of variance followed by post hoc Sidak tests. **(F)** Effects of intraperitoneal injections of saline, fluoxetine (10 mg/kg), or d-Fen (3 mg/kg) on 2.5-hour HFD intake in WT or 2C-null mice exposed to continuous chow/HFD feeding. *n* = 6 or 7 per group. Results are shown as mean ± SEM. ns, not significant.

We used an established protocol (29), intermittent HFD exposure, to train mice to develop binge-like eating behavior (Figure 1A). After an initial 1-week training, these intermittent mice developed a repetitive eating behavior that mimics many characteristics of binge eating in humans. For example, intermittent mice ate a large amount of HFD over a short period of time (between 11:00 am and 1:30 pm) (Figure 1B). A preference for high palatability was clearly observed in these mice, with about 0.8 g HFD intake compared with minimal chow intake during this 2.5-hour period (Figure 1B). In contrast, control mice, those exposed either to continuous chow/HFD feeding or to continuous chow feeding, ate minimal amounts HFD and/or chow during the same period (Figure 1B). We confirmed that these intermittent mice were satiated during this binging period, because when these mice were provided only chow, instead of chow and HFD, they ate minimal food, which was comparable to that of control mice (Figure 1C). Finally, the binge-like eating seen in intermittent mice was repetitive and persisted for at least seven cycles without any evidence of habituation (Figure 1D), which is similar to the recurrent binge episodes in patients. Thus, we measured the 2.5-hour HFD intake in intermittent mice to assess binge-like eating behavior in the following studies.

We first showed that i.p. injections of fluoxetine (10 mg/kg) or d-Fen (3 mg/kg) significantly suppressed binge-like eating in WT mice (Figure 1E). However, in a loxed transcription blocker (loxTB) 5-HT_2C_R (2C-null) mouse line lacking functional 5-HT_2C_Rs globally (32, 33), neither fluoxetine nor d-Fen inhibited binge-like eating (Figure 1E). These results indicate that the inhibitory effects of these 5-HT drugs on binge-like eating are mediated in part through the 5-HT_2C_R, one of the 14 known receptors for 5-HT in mammals. As a non-binge-eating control group, another cohort of WT and 2C-null mice were subjected to the continuous chow/HFD exposure. The 2.5-hour HFD intake in these “continuous” mice was minimal, and importantly, no significant effects of fluoxetine and d-Fen were observed during this period in either WT or 2C-null mice (Figure 1F).

### 5-HT Neurons Innervate DA Neurons

**Figure 2:**
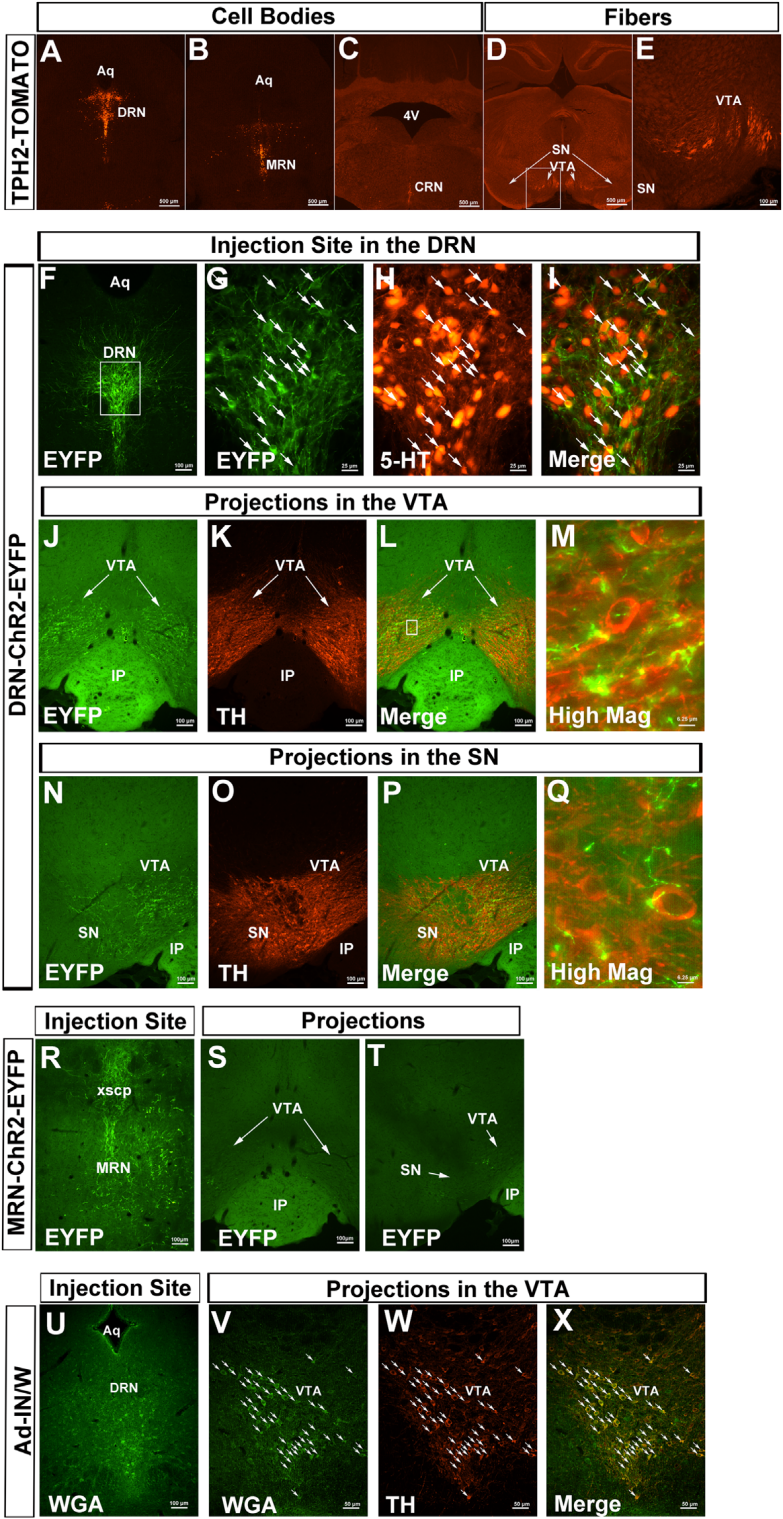
5-Hydroxytryptamine (5-HT) neurons synapse on dopamine neurons. **(A–E)** TOMATO-labeled cell bodies **(A–C)** and TOMATO-positive fibers **(D, E)** in the brain of tryptophan hydroxylase-2 (TPH2)-CreER/Rosa26-tdTOMATO mice (after tamoxifen induction). **(E)** Higher magnification of the white box in **(D). (F–Q)** AAV-EF1α-DIO hChR2(H134R)-EYFP was injected into the dorsal raphe nuclei (DRN) of TPH2-CreER mice (with tamoxifen induction). **(F)** A low-magnification image for the injection site showing enhanced yellow fluorescent protein (EYFP)-labeled cell bodies and fibers within the DRN; **(G–I)** High-magnification images of the white box in **(F),** showing EYFP exclusively colocalized with 5-HT (arrow-pointed neurons). **(J–M)** The projections in the ventral tegmental area (VTA) are indicated, showing EYFP-labeled fibers in the close proximity with tyrosine hydroxylase (TH)-labeled cell bodies. **(N–Q)** The fewer projections in the substantia nigra (SN) are indicated. **(R–T)** AAV-EF1α-DIO hChR2(H134R)-EYFP was injected into the median raphe nuclei (MRN) of TPH2-CreER mice (with tamoxifen induction). **(R)** The injection site is indicated, showing EYFP-labeled cell bodies and fibers within the MRN; **(S, T)** indicate scarce EYFP-labeled fibers in the ventral tegmental area (VTA) and SN. **(U–X)** Ad-IN/W virus was injected into the DRN of TPH2-CreER mice (with tamoxifen induction). **(U)** The injection site is indicated, showing wheat germ agglutinin (WGA)-labeled neurons within the DRN; **(V–X)** indicate anterograde WGA signals in the VTA, largely colocalized with TH. Scale bars are indicated in each image. Scale bars = 500 μm in **(A-D)**, 100 μm in **(E, F, J–L, N–P, R–T, U)**, 50 μm in **(V–X)**, 25 μm in **(G–I)**, and 6.25 μm in **(M, Q)**. Arrows in **(N–P)** point to double-labeled neurons. 4V, fourth ventricle; AAV, adeno-associated virus; Aq, aqueduct; ChR2, channelrhodopsin-2; CRN, caudal raphe nuclei; IP, interpeduncular nucleus; xscp, decussation of superior cerebellar peduncle.

VTA DA neurons (DA^VTA^) coexpress 5-HT_2C_Rs (34). Given that dysfunctions of brain DA system are implicated in the development of binge eating in humans (18, 19), we speculated that 5-HT_2C_Rs expressed by DA neurons may be involved in the inhibitory effects of 5-HT drugs on binge-like eating. To test this possibility, we first used TPH2-CreER mice that express tamoxifen-inducible Cre recombinase exclusively in TPH2-expressing neurons. We crossed TPH2-CreER mice with Rosa26-tdTOMATO mice to generate TPH2-CreER/Rosa26-tdTOMATO mice. After tamoxifen inductions (4 mg/mouse, i.p.) in these mice, we detected abundant TOMATO-labeled cell bodies in the DRN, median raphe nuclei (MRN), and caudal raphe nuclei (Figure 2A–C). We further observed intensive TOMATO-positive terminals in the VTA, with fewer terminals in the adjacent SN (Figure 2D, E). These results suggest that 5-HT neurons project to the VTA as well as SN. To directly determine which subsets of 5-HT neurons project to the VTA/SN, we stereotaxically injected Cre-dependent AAV expressing channelrhodopsin-2 (ChR2) enhanced yellow fluorescent protein (EYFP) into the DRN of TPH2-CreER mice to effectively infect 5-HT^DRN^ neurons (Figure 2F–I), and observed ChR2-EYFP–labeled fibers in the VTA and SN, which were in close proximity with tyrosine hydroxylase-positive neurons (Figure 2J–Q). When this AAV virus was injected in the MRN of TPH2-CreER mice (Figure 2R), only scarce ChR2-EYFP–labeled fibers were observed in the VTA/SN (Figure 2S, T). These results indicate that the VTA/SN receive projections from 5-HT^DRN^ neurons and, to a lesser extent, from 5-HT^MRN^ neurons.

We then stereotaxically injected Ad-IN/W (35) into the DRN of TPH2-CreER mice. On tamoxifen induction, these mice expressed green fluorescent protein–tagged wheat germ agglutinin ([WGA], which is an anterograde trans-synaptic tracer) specifically in 5-HT^DRN^ neurons (Figure 2U). As expected, WGA immunoreactivity was observed in brain regions that are innervated by 5-HT neurons (36), including the red nucleus, the arcuate nucleus, and the paraventricular nucleus of the hypothalamus (Supplemental Figure S1A–F). Importantly, we also detected WGA-labeled neurons in the VTA, the majority of which were confirmed to be DA neurons with costaining of tyrosine hydroxylase (Figure 2V–X). Collectively, these data established a neural circuit in which 5-HT^DRN^ neurons innervate DA^VTA^ neurons.

### 5-HT_2C_R in DA Neurons Mediates Effects of Fluoxetine and d-Fen on Binge-like Eating in Mice

We crossed DAT-CreER mice with Rosa26-tdTOMATO mice to generate DAT-CreER/Rosa26-tdTOMATO mice and confirmed that Cre-induced TOMATO expression was exclusively located in DA neurons in the VTA and SN (Supplemental Figure S2A–C), but not in other brain regions (Supplemental Figure S2D–F). Importantly, DAT-CreER/Rosa26-tdTOMATO mice without tamoxifen induction do not show any leaking Cre activity (Supplemental Figure S2G).

**Figure 3:**
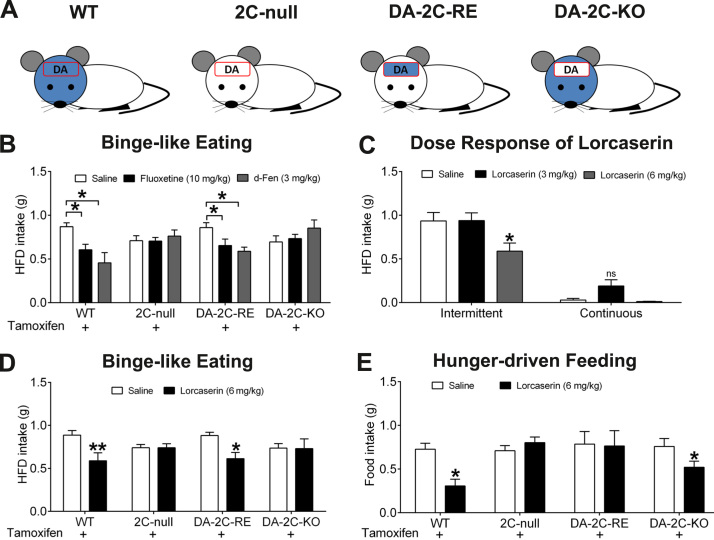
5-Hydroxytryptamine 2C receptors (5-HT_2C_Rs) in dopamine (DA) neurons mediate effects of 5-HT drugs to inhibit binge-like eating. **(A)** Illustration of 5-HT_2C_R expression pattern in wild-type (WT), 2C-null, DA-2C-RE or DA-2C-KO mice. Blue indicates the distribution of 5-HT_2C_Rs (note that 5-HT_2C_Rs are reported to be exclusively in the brain); the red box indicates DA neural population. **(B)** Effects of intraperitoneal (i.p.) injections of saline, fluoxetine (10 mg/kg), or d-fenfluramine (d-Fen) (3 mg/kg) on binge-like eating (2.5-hour high-fat diet [HFD] intake) in four groups of mice that all received tamoxifen and were exposed to intermittent HFD feeding. *n* = 6 to 9 per group. Results are shown as mean ± SEM. ^*^p < .05 between drug treatment and saline treatment in the same genotype in two-way analyses of variance (ANOVAs) followed by post hoc Sidak tests. **(C)** Effects of i.p. injections of saline, lorcaserin (3 mg/kg), or lorcaserin (6 mg/kg) on 2.5-hour HFD intake in WT mice exposed to intermittent HFD feeding or continuous chow/HFD feeding. *n* = 10 to 12 per group. Results are shown as mean ± SEM. ^*^p < .05 between drug treatment and saline treatment in the same feeding paradigm in two-way ANOVAs followed by post hoc Sidak tests. **(D)** Effects of i.p. injections of saline or lorcaserin (6 mg/kg) on binge-like eating (2.5-hour HFD intake) in four groups of mice that all received tamoxifen and were exposed to intermittent HFD feeding. *n* = 7 to 10 per group. Results are shown as mean ± SEM. ^*^p < .05 and ^**^p < .01 between lorcaserin treatment and saline treatment in the same genotype in two-way ANOVAs followed by post hoc Sidak tests. **(E)** Effects of i.p. injections of saline or lorcaserin (6 mg/kg) on 2-hour refeeding after an overnight fast in four groups of mice that all received tamoxifen and were maintained on chow diet. *n* = 7 to 15 per group. Results are shown as mean ± SEM. ^*^p < .05 between lorcaserin treatment and saline treatment in the same genotype in two-way ANOVAs followed by post hoc Sidak tests. KO, knockout; ns, not significant; RE, re-expression.

We then crossed DAT-CreER mice to 2C-null (loxTB-5-HT_2C_R) mice to generate littermates of WT mice, 2C-null mice, and DA-2C-RE mice (those carrying both mutations). In DA-2C-RE mice, tamoxifen-induced Cre recombinase is expected to remove the loxTB cassette and reactivate expression of endogenous 5-HT_2C_Rs only in DA neurons (33). As predicted (Figure 3A), WT mice express 5-HT_2C_R messenger RNAs (detected by reverse transcriptase polymerase chain reaction) in all brain regions examined, including the amygdala, frontal cortex, hypothalamus, and VTA, whereas 2C-null mice do not express 5-HT_2C_R messenger RNAs in any of these regions (Supplemental Figure S2H). DA-2C-RE mice express 5-HT_2C_R messenger RNAs in the VTA, but not in other brain regions (Supplemental Figure S2H).

We showed that both fluoxetine and d-Fen significantly suppressed binge-like eating in WT mice, but these effects were blunted in 2C-null mice (Figure 3B). Importantly, we showed that the antibinge effects of fluoxetine and d-Fen were largely restored in DA-2C-RE mice (Figure 3B). These data indicate that 5-HT_2C_Rs expressed by DA neurons alone are sufficient to restore antibinge effects of 5-HT drugs in mice.

We have recently generated a lox-5-HT_2C_R mouse, in which the critical coding region of the 5-HT_2C_R gene was flanked by two loxP sites and can be deleted in Cre-positive cells (37). Here we crossed DAT-CreER mice with lox-5-HT_2C_R mice to generate DA-2C-KO mice (those carrying both mutations). In DA-2C-KO mice, tamoxifen-induced Cre recombinase resulted in deletion of endogenous 5-HT_2C_Rs only in DA neurons (as illustrated in Figure 3A). Interestingly, we found that both fluoxetine and d-Fen failed to impact binge-like eating in DA-2C-KO mice (Figure 3B). These data indicate that 5-HT_2C_Rs expressed by DA neurons are required to mediate antibinge effects of 5-HT medications.

### Lorcaserin Acts on 5-HT_2C_Rs in DA Neurons to Inhibit Binge-like Eating in Mice

A selective 5-HT_2C_R agonist, lorcaserin (Arena Pharmaceuticals, San Diego, CA), was approved by the Food and Drug Administration for obesity treatment in 2012 and has been prescribed to obese patients at 10 mg (twice a day). Here we showed that lorcaserin at 6 mg/kg, but not at 3 mg/kg, significantly inhibited binge-like eating (Figure 3C). These results indicate that lorcaserin is effective in suppressing binge-like eating behavior in mice. We further tested antibinge effects of lorcaserin (6 mg/kg) in WT, 2C-null, DA-2C-RE, and DA-2C-KO mice (all of which first received tamoxifen injections and were then subjected to the intermittent HFD exposure). Similar to fluoxetine and d-Fen, lorcaserin significantly suppressed HFD intake in WT mice, but these effects were attenuated in both in 2C-null mice and in DA-2C-KO mice (Figure 3D). Importantly, re-expression of 5-HT_2C_Rs only in DA neurons in DA-2C-RE mice was sufficient to restore antibinge effects of lorcaserin close to the level observed in WT mice (Figure 3D). Together, these results highlight the 5-HT_2C_R in DA neurons as a key mediator for the inhibitory effects of lorcaserin on binge-like eating in mice.

### 5-HT_2C_Rs in DA Neurons Do Not Mediate Effects of Lorcaserin on Hunger-Driven Food Intake in Mice

**Figure 4:**
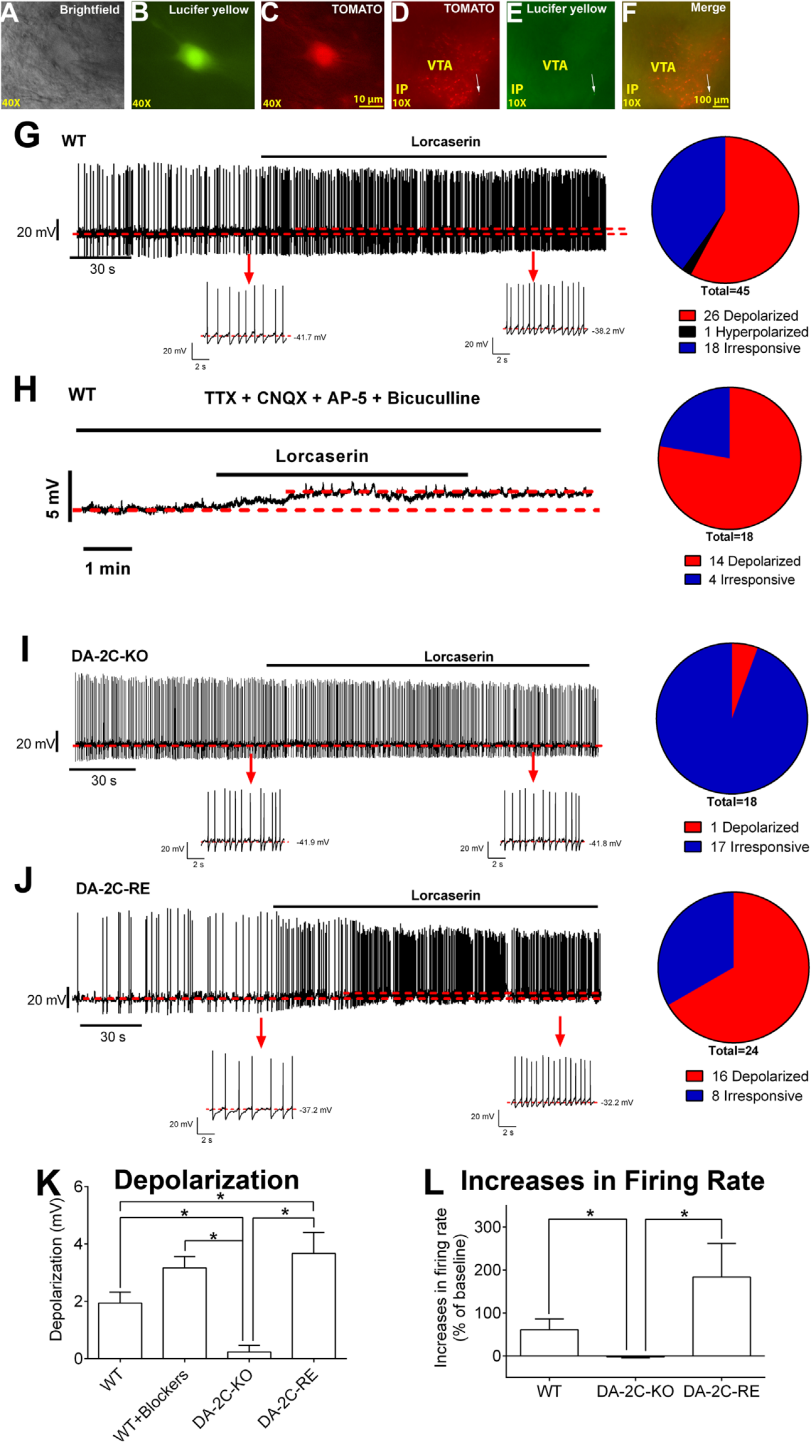
Lorcaserin activates dopamine ventral tegmental area (DA^VTA^) neurons via 5-hydroxytryptamine 2C receptor–mediated mechanisms. **(A–C)** Brightfield **(A),** fluorescent with fluorescein isothiocyanate filter **(B),** and with tetramethylrhodamine filter **(C)** illuminations of a targeted DA^VTA^ neuron. Panel **(B)** shows the lucifer yellow dye injected into the recorded neuron; **(C)** shows TOMATO signal. Scale bars = 10 µm. **(D–F)** Post hoc identification of the recorded neuron within the VTA in the fixed brain slice. Panel **(D)** shows TOMATO-labeled DA neurons in the VTA; **(E)** shows the recorded neurons filled with the lucifer yellow dye; and **(F)** shows the merge of **(D)** and **(E)**. Scale bars =100 µm. **(G, H)** Representative traces before and after lorcaserin treatment (30 µM, bath perfusion) in DA^VTA^ neurons from wild-type (WT) mice in the absence of **(G)** or the presence of 1 μM tetrodotoxin (TTX), 30 μM CNQX, 30 μM AP5, and 50 μM bicuculline **(H). (I, J)** Representative traces before and after lorcaserin treatment (30 µM, bath perfusion) in DA^VTA^ neurons from DA-2C-KO mice **(I)** and DA-2C-RE mice **(J).** The upper panels show the long firing traces; the lower panels show zoom-in traces before and after lorcaserin treatment. The pie graphs in **(G–J)** represent the number (percentage) of neurons with depolarization, hyperpolarization, or no response. **(K)** Magnitude of depolarization induced by lorcaserin in various groups. *n* = 18 to 45 per group. Results are shown as mean ± SEM. ^*^p < .05 and ^**^p < .01 by unpaired *t* tests. **(M)** Changes in firing rate induced by lorcaserin in various groups. *n* = 13 to 15 per group. Results are shown as mean ± SEM. ^*^p < .05 by unpaired *t* tests. IP, interpeduncular nucleus; KO, knockout; RE, re-expression.

Agonists of the 5-HT_2C_R, including lorcaserin (3–24 mg/kg), strongly suppress fast-induced refeeding in animals (38), indicating that 5-HT_2C_R signals also regulate feeding behavior driven by hunger. To further determine whether 5-HT_2C_Rs in DA neurons are also involved in hunger-driven feeding, we tested effects of lorcaserin on fast-induced refeeding in another cohort of mice. We found that lorcaserin (6 mg/kg, i.p.) significantly inhibited 2-hour food intake in WT mice that had fasted overnight (Figure 4E). The lorcaserin-induced anorexia was blocked in 2C-null (Figure 3E), confirming that effects of lorcaserin on hunger-driven feeding are mediated through 5-HT_2C_Rs. Importantly, re-expression of 5-HT_2C_Rs in DA neurons (in DA-2C-RE mice) failed to rescue the lorcaserin-induced anorexia (Figure 4E). In addition, similarly as for WT mice, DA-2C-KO mice with 5-HT_2C_Rs selectively deleted in DA neurons responded to lorcaserin with significantly decreased food intake (Figure 3E). Similar patterns were observed at 4 and 6 hours after refeeding (Supplemental Figure S4). Together, our results indicate that 5-HT_2C_Rs mediate lorcaserin actions to suppress hunger-driven feeding behavior, but these effects are not dependent on 5-HT_2C_Rs expressed by DA neurons.

### Lorcaserin Activates DA Neurons via 5-HT_2C_R–Mediated Mechanisms

To delineate the cellular mechanisms by which 5-HT_2C_Rs influence DA neuron activity, we visualized DA neurons (Figure 4A–F) with and without altered 5-HT_2C_R expression through intercrossing mice with a Rosa26-tdTOMATO allele with DAT-CreER, DA-2C-RE, and DA-2C-KO mice, respectively. We first assessed the basic electrophysiological properties of identified DA^VTA^ neurons from WT, DA-2C-KO, and DA-2C-RE mice. We found that the input resistances of DA neurons from DA-2C-RE mice were significantly lower than DA neurons from WT mice, whereas there was no difference between WT and DA-2C-KO mice (Supplemental Figure S10D, E). Resting membrane potential was comparable among all genotypes (Supplemental Figure S10F); similar proportions of DA neurons from all genotypes fired spontaneously (Supplemental Figure S10G), and baseline firing rate neurons were comparable among the 3 genotypes (Supplemental Figure S10H).

**Figure 5:**
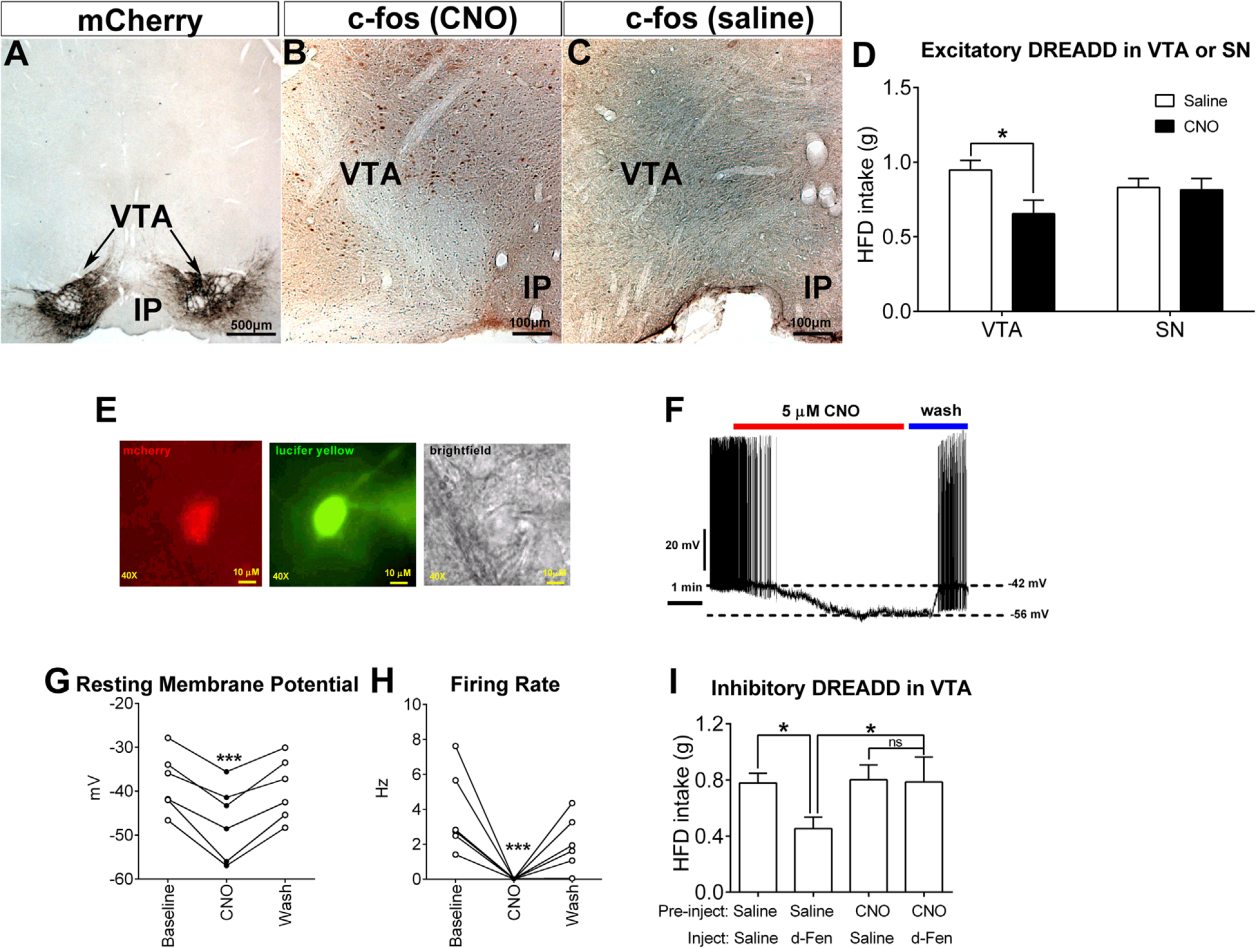
5-Hydroxytryptamine drugs inhibit binge-like eating via stimulating dopamine ventral tegmental area (DA^VTA^) neurons. **(A–C)** Validation of AAV-hM3Dq-mCherry-mediated activation of DA neurons in the VTA. **(A)** mCherry immunoreactivity after injections of excitatory AAV-hM3Dq-mCherry into the VTA of DAT-CreER mice. **(B, C)** c-fos immunoreactivity in the VTA 90 minutes after injections of 1 mg/kg clozapine N-oxide (CNO) **(B)** or saline **(C).** Scale bars = 500 μm **(A)** and 100 μm (**B, C). (D)** Effects of saline or CNO (1 mg/kg, i.p.) on binge-like eating in DAT-CreER mice receiving excitatory AAV-hM3Dq-mCherry infection in the VTA or in the substantia nigra (SN). Results are shown as mean ± SEM. ^*^p < .05 by unpaired *t* tests. **(E)** Fluorescence of mCherry and lucifer yellow, and brightfield illuminations of a targeted DA^VTA^ neuron infected with inhibitory AAV-hM4Di-mCherry. Scale bars = 10 µm. **(F)** Representative electrophysiological response to CNO (5 µM, bath) in DA^VTA^ neurons infected with inhibitory AAV-hM4Di-mCherry. **(G, H)** Summary changes in resting membrane potential **(G)** and in firing rate **(H)** in DA^VTA^ neurons infected with inhibitory AAV-hM4Di-mCherry. *n* = 6/group. ^***^p < .001 by paired *t* tests. **(I)** Effects of saline/CNO (3 mg/kg, i.p.) followed by saline or d-fenfluramine (d-Fen) (3 mg/kg, i.p.) on binge-like eating measured in DAT-CreER mice receiving inhibitory AAV-hM4Di-mCherry infection in the VTA. *n* = 6 or 7 per group. Results are shown as mean ± SEM. ^*^p < .05 in two-way analyses of variance followed by post hoc Sidak tests. AAV, adeno-associated virus; DAT, dopamine transporter; DREADD, designer receptors exclusively activated by designer drugs; HFD, high-fat diet; IP, interpeduncular nucleus; i.p., intraperitoneal.

Then we examined effects of lorcaserin on these DA neurons from 3 genotypes. In WT mice, a portion of DA neurons (57.8%, 26 of 45) responded to lorcaserin treatment (30 µM, bath perfusion) with depolarization (see Figure 5G for representative traces); one DA neuron from WT mice (2.22%) was hyperpolarized by 2.53 mV; the rest of DA neurons (40.0%, 18 of 45) were irresponsive (≤2 mV changes in resting membrane potential) (Table 1). We also showed that lorcaserin depolarized 77.8% (14 of 18) DA neurons (see Figure 4H for representative traces) in the presence of tetrodotoxin and a cocktail of fast synaptic inhibitors (bicuculline, AP5 [DL-2-amino-5-phosphonovaleric acid; Sigma-Aldrich, St. Louis, MO], and CNQX [6-cyano-7-nitroquinoxaline-2,3-dione; Sigma-Aldrich]), indicating that this lorcaserin-induced depolarization was mediated through a direct action of lorcaserin on these DA neurons. Supporting this possibility, we showed that the majority of DA neurons from DA-2C-KO mice (94.44%, 17 of 18) were irresponsive to lorcaserin (Figure 4I and Table 1). Importantly, in DA-2C-RE mice with 5-HT_2C_Rs re-expressed only in DA neurons, we found that lorcaserin depolarized 66.67% (16 of 24) DA neurons (see Figure 4J for representative traces). We further compared the magnitude of depolarization in various groups and found that effects of lorcaserin in WT neurons were significantly diminished in DA-2C-KO neurons, but this lorcaserin-induced depolarization was rescued in DA-2C-RE neurons (Figure 4K). Interestingly, the depolarization in DA-2C-RE neurons was significantly stronger than that in WT neurons (Figure 4K). Lorcaserin increased the firing rate in WT neurons, an effect that was blocked in DA-2C-KO neurons, but restored in DA-2C-RE neurons (Figure 4L). Together, these results indicate that lorcaserin activates DA^VTA^ neurons via 5-HT_2C_R-mediated mechanisms.

**Table 1:** Number of DA^VTA^ Neurons That Responded to Lorcaserin

Genotype	Total	Depolarized	Hyperpolarized	Irresponsive
WT	45	26 (57.78)^a^	1 (2.22)	18 (40.00)^a^
WT + Blockers	18	14 (77.78)^a^	0 (0)	4 (22.22)^a^
DA-2C-KO	18	1 (5.56)	0 (0)	17 (94.44)
DA-2C-RE	24	16 (66.67)^a^	0 (0)	8 (33.33)^a^

Values are count or *n* (% of count in total cell). Depolarization was defined as >2 mV elevations in resting membrane potential within 3 min after lorcaserin application; hyperpolarization was defined as >2 mV reductions in resting membrane potential within 3 min after lorcaserin application; other neurons were defined as irresponsive.

DA, dopamine; KO, knockout; RE, re-expression; VTA, ventral tegmental area; WT, wild type.

a *p* < .01 vs. DA-2C-KO group in chi-square test.

### 5-HT Inhibits Binge-like Eating by Activating DA Neurons

DAT-CreER mice (12 weeks) received stereotaxic injections of the excitatory DREADD virus (designer receptor, AAV-hM3Dq-mCherry) into both sites of VTA. Upon tamoxifen injection (4 mg, i.p.), hM3Dq-mCherry was exclusively expressed in DA^VTA^ neurons, as confirmed by post hoc immunostaining for mCherry (Figure 5A). Designer drug CNO administration (1 mg/kg, i.p.) selectively activates DA neurons, as demonstrated by induction of c-fos immunoreactivity in the VTA in post hoc analyses (Figure 5B). As a negative control, we confirmed that saline administration did not induce c-fos immunoreactivity in the VTA (Figure 5C). CNO administration significantly inhibited binge-like eating when compared with saline administration (Figure 5D). As a further control of the specificity of function of DA^VTA^ neurons, the SN was targeted in another group of mice (see Supplemental Figure S11 for validation). Unlike activation of DA^VTA^ neurons, discrete activation of DA^SN^ neurons did not affect binge-like eating (Figure 5D).

Next, a group of DAT-CreER mice (8 weeks) received stereotaxic injections of the inhibitory DREADD virus AAV-hM4Di-mCherry into the VTA followed by treatment with tamoxifen. Electrophysiological recordings revealed that CNO treatment (5 µM) rapidly and robustly inhibited mCherry-labeled DA^VTA^ neurons (Figure 5E–H). Another group of DAT-CreER mice (12 weeks) received stereotaxic bilateral injections of AAV-hM4Di-mCherry into the VTA. These mice then received pretreatment with saline or CNO (3 mg/kg, 60 minutes before), followed by treatment with saline or d-Fen (3 mg/kg, 30 minutes before), and binge-like eating was measured 30 minutes later. Although d-Fen significantly inhibited binge-like eating in mice pretreated with saline, in mice pretreated with CNO (DA^VTA^ neurons inhibited), d-Fen failed to suppress binge-like eating (Figure 5I). These results further support that 5-HT compounds inhibit binge-like eating through stimulating DA^VTA^ neural activities.

## Discussion

In the current study, we provide evidence to support that a 5-HT→DA neural circuit exerts inhibitory effects on binge-like eating behavior in mice. We used ChR2 and WGA anterograde tracing approaches to provide complementary evidence that 5-HT neurons primarily originating from the DRN project to DA^VTA^ neurons. Consistent with earlier electron microscopic observations in rat brains (39, 40) and a recent retrograde tracing study in mice (28), our results provided neuroanatomic evidence for the presence of a midbrain 5-HT→DA neural circuit.

Previous observations regarding effects of 5-HT on DA neural activity have been controversial. On one hand, microinfusion of 5-HT directly into the VTA of rats enhances DA release (41), suggesting an excitatory effect of 5-HT on DA neural activity. On the other hand, it was reported that the firing rate of putative DA^VTA^ neurons in rats can be inhibited by intravenous administration of a 5-HT_2C_R agonist (RO600175) (42, 43). This discrepancy may result from nonspecificity of 5-HT compounds, different delivery routes, and perhaps unidentified neural populations in these studies. Here we used slice electrophysiology to directly record effects of the selective 5-HT_2C_R agonist (lorcaserin) on neural activities of identified DA^VTA^ neurons. Lorcaserin depolarized a large portion of DA^VTA^ neurons and increased their firing rate. The lorcaserin-induced depolarization persisted in the presences of tetrodotoxin and glutamate/gamma-aminobutyric acid receptor antagonists, confirming a direct effect of lorcaserin on DA^VTA^ neurons. More importantly, lorcaserin-induced activation was almost abolished in mice lacking 5-HT_2C_Rs only in DA neurons, but was rescued in mice with 5-HT_2C_Rs re-expressed only in DA neurons. Together, these studies support that 5-HT activates at least a portion of DA^VTA^ neurons through 5-HT_2C_R-mediated mechanisms. Notably, DA neurons from DA-2C-RE mice are even more responsive to lorcaserin treatment than those from WT mice, suggesting that in WT mice, lorcaserin may also exert an inhibitory tone on DA neurons via indirect mechanisms. Supporting this possibility, DA^VTA^ neurons are known to receive tonic inhibitory inputs from gamma-aminobutyric acidergic interneurons (44) and gamma-aminobutyric acidergic neurons express 5-HT_2C_Rs (34).

We found that drugs that enhance brain 5-HT content (fluoxetine and d-Fen) and the selective 5-HT_2C_R agonist (lorcaserin) effectively suppressed binge-like eating in WT mice. These effects were attenuated in 2C-null mice lacking 5-HT_2C_Rs globally or in DA-2C-KO mice that lack 5-HT_2C_Rs only in DA neurons. Furthermore, re-expression of 5-HT_2C_Rs only in DA neurons in DA-2C-RE mice restored antibinge effects of these 5-HT medications close to the level observed in WT mice. Thus, these results highlight the importance of 5-HT_2C_Rs in DA neurons in the regulation of binge-like eating. The functional relevance of this 5-HT→DA neural circuit was further examined using both excitatory and inhibitory chemogenetic approaches. We demonstrated that activation of DA^VTA^ neurons inhibits binge-like eating, simulating effects of 5-HT drugs, whereas inhibition of DA^VTA^ neurons blocks antibinge effects of 5-HT drugs. Collectively, our results indicate that 5-HT activates DA^VTA^ neurons, which results in inhibition of binge-like eating. It is worth mentioning that binge-like eating is only affected by activities of DA neurons in the VTA, but not by those in the SN. Consistently, it has been reported that electric stimulation of the nucleus accumbens, a region receiving intensive dopaminergic inputs from the VTA, potently inhibits binge-like eating in mice (24). On the other hand, electric stimulation of the dorsal striatum, a region receiving dopaminergic inputs from the SN, has been reported to have no effect on binge-like eating (24).

Identification of the action target for 5-HT to inhibit binge-like eating in animals may facilitate development of more selective and safer therapeutic interventions for binge eating in humans. We revealed that the majority of antibinge benefits induced by fluoxetine and d-Fen are mediated by the 5-HT_2C_R. Providing a rationale for the clinical adoption of a 5-HT_2C_R agonist for the treatment of binge eating, we report that the Food and Drug Administration–approved drug lorcaserin (used for weight management) effectively suppresses binge-like eating in mice. Furthermore, we observed that these effects are largely mediated by 5-HT_2C_Rs in DA neurons. Together, our results provide compelling preclinical evidence for lorcaserin for the treatment of binge eating.

Both d-Fen (45, 46) and lorcaserin (38) suppress hunger-driven feeding through 5-HT_2C_R-mediated mechanisms. Thus, an important question is whether effects of 5-HT_2C_Rs in DA neurons on binge-like eating are dependent on regulations on hunger-driven feeding. To distinguish binge-like eating from hunger-driven feeding behavior, we used a fast-induced refeeding paradigm to directly examine effects of lorcaserin on hunger-driven food intake. Lorcaserin suppressed fast-induced refeeding in WT mice, but not in 2C-null mice. These findings were consistent with the known effects of lorcaserin (38) and the established roles of 5-HT_2C_Rs in the regulation of hunger-driven feeding (33, 47). However, the lorcaserin-induced anorexia in this fast-refeeding paradigm was not affected by deletion of 5-HT_2C_Rs in DA neurons, nor was it rescued by restoration of 5-HT_2C_Rs only in DA neurons. Such findings indicate that 5-HT_2C_Rs in DA neurons are not involved in regulation of hunger-driven feeding behavior.

In summary, our data support a model in which 5-HT stimulates DA neural activity through a 5-HT_2C_R-mediated mechanism, and activation of this midbrain 5-HT→DA neural circuit effectively inhibits binge-like eating behavior in mice. We identified the 5-HT_2C_R population in DA neurons as one potential target for antibinge therapies. Finally, we provided preclinical evidence that the Food and Drug Administration–approved 5-HT_2C_R agonist, lorcaserin, can be repurposed for the treatment of binge eating.
