# Supplementary material for: Activation of Serotonin 2C Receptors in Dopamine Neurons Inhibits Binge-like Eating in Mice
Source: Biol Psychiatry. 2017 May 1;81(9):737–47. doi: 10.1016/j.biopsych.2016.06.005 (PMC5148733; doi:10.1016/j.biopsych.2016.06.005)
Supplement: Supplementary file 1 — Supplementary material [file mmc1.pdf]

Activation of Serotonin 2C Receptors in Dopamine Neurons Inhibits Binge-like Eating in Mice

Supplemental Information

Supplemental Methods

Mice

We crossed male C57Bl6 mice with female mice that were heterozygous for the loxTB-5-HT_{2C}R allele (1). This cross generated male wild type (WT) mice and mutant mice that carry one loxTB-5-HT_{2C}R allele. Since the gene encoding 5-HT_{2C}Rs is located on the X chromosome and male mice only carry one allele, these male mutant mice were actually 2C-null mice. These mice were used for experiments in Figure 1D and 1E.

In addition, we crossed male DAT-CreER (Jackson Laboratory, #016583) transgenic mice and female mice that were heterozygous for 2C-null (loxTB-5-HT_{2C}R) allele. This cross produced male WT, 2C-null, and DA-2C-RE (DAT-CreER/loxTB-5-HT_{2C}R) mice. In parallel, male DAT-CreER mice were crossed to female mice that were heterozygous for the lox-5-HT_{2C}R allele (2). This cross generated WT and DA-2C-KO (DAT-CreER/lox-5-HT_{2C}R) mice. At 8 weeks of age, all mice received intraperitoneal (i.p.) injections of tamoxifen (4 mg/mouse). In DA-2C-RE mice, tamoxifen induced Cre activity to re-express 5-HT_{2C}Rs only in DA neurons; while in DA-2C-KO mice, tamoxifen-induced Cre activity led to deletion of 5-HT_{2C}Rs only in DA neurons. Tamoxifen was also injected into the WT and 2C-null mice in order to exclude effects of tamoxifen itself. These mice were used for the measurements of feeding, anxiety, depression and physical activities (Figures 3, S3-S9). WT mice obtained from two different crosses were pooled in these studies.

For electrophysiological studies (Figures 5 and S10), we crossed Rosa26-tdTOMATO allele onto DAT-CreER, DA-2C-RE, and DA-2C-KO mice, respectively. These crosses generated WT, DAT-2C-RE and DA-2C-KO mice with TOMATO selectively expressed in DA neurons (after tamoxifen inductions). Note that since expression of TOMATO requires Cre activity in DA neurons, it is impossible to generate 2C-null mice with TOMATO expressed in DA neurons.

In addition, some of DAT-CreER/Rosa26-tdTOMATO mice were used for validation of Cre activity (Figure S2). We also crossed DAT-CreER mice with C57Bl6 mice to generate DAT-CreER mice. These mice were used for DREADD studies (Figures 5 and S11).

To visualize 5-HT neuron cell bodies and fibers (Figure 2A), TPH2-CreER (Jackson Laboratory, #016584) transgenic mice were crossed to Rosa26-tdTOMATO mice to generate TPH2-CreER/Rosa26-tdTOMATO mice. We have previously shown that these mice express TOMATO exclusively in 5-HT neurons upon tamoxifen induction (3). This cross also generated and TPH2-CreER mice without the Rosa26-tdTOMATO allele. These TPH2-CreER mice were used in ChR2 and Ad-IN/W tracing experiments (Figures 2 and S1).

All the breeders have been backcrossed to C57Bl6 background for more than 12 generations. In addition, some C57Bl6 mice were purchased from the mouse facility of Baylor College of Medicine. Mice were housed in a temperature-controlled environment in groups of two to five at 22˚C-24˚C using a 12 hr light/12 hr dark cycle. The mice were fed standard chow (6.5% fat, #2920, Harlan-Teklad, Madison, WI) until training and assessment of binge-like eating behavior. Water was provided ad libitum.

Visualization of 5-HT Cell Bodies and Fibers

Twelve-week old TPH2-CreER/Rosa26-tdTOMATO mice (male or female) received i.p. injections of tamoxifen (4 mg/mouse). One week after tamoxifen induction, mice were perfused with 10% formalin, and brain sections were cut at 25 μm (5 series). The sections were directly mounted on glass slides and TOMATO red fluorescence images were analyzed using a Leica DM5500 fluorescence microscope with OptiGrid structured illumination configuration.

ChR2 Anterograde Tracing

To determine which sub-sets of 5-HT neurons project to the VTA/SN, twelve-week old TPH2-CreER mice were anesthetized by isofluorane and received stereotaxic injections of Cre-dependent AAV expressing ChR2-EYFP (AAV-EF1α-DIO hChR2(H134R)-EYFP) into the DRN (200 nl; 4.6 mm posterior, 0 mm lateral and 3.5 mm ventral to the Bregma, based on Franklin & Paxinos Mouse Brain Atlas) or the MRN (200nl; 4.6 mm posterior, 0 mm lateral and 4.4 mm ventral to the Bregma, based on Franklin & Paxinos Mouse Brain Atlas). The mice also received i.p. injections of tamoxifen (4 mg/mouse, i.p.). One week after injections, mice were perfused with 10% formalin, and brain sections were cut at 25 μm (5 series). The sections were directly mounted on glass slides and EYFP fluorescence images were analyzed using a Leica DM5500 fluorescence microscope with OptiGrid structured illumination configuration.

Ad-IN/W Anterograde Tracing

In order to confirm that DRN 5-HT neurons synapse on DA neurons, twelve-week old TPH2-CreER mice were anesthetized by isoflurane and received stereotaxic injections of Ad-IN/W (4) into the DRN (200 nl; 4.6 mm posterior, 0 mm lateral and 3.5 mm ventral to the Bregma, based

on Franklin & Paxinos Mouse Brain Atlas). The mice also received i.p. injections of tamoxifen (4 mg/mouse, i.p.). In these mice, Ad-IN/W induced expression of GFP-WGA only in DRN 5-HT neurons; WGA anterogradely travelled along the 5-HT fibers, passed the synapse, and filled the downstream neurons that were innervated by 5-HT terminals. Three days after injections, mice were perfused with 10% formalin, and brain sections were cut at 25 μm (5 series). The sections were incubated at room temperature in primary goat anti-WGA antibody (1:1000, #AS-2024, VectorLabs) overnight, followed by the secondary donkey anti-goat Alexa Fluor 488 (1:500; #A-11055, Invitrogen) for 1.5 hours. Then, the sections were incubated in the primary rabbit anti-TH antibody (1:1000, #AB-152, Millipore) overnight, followed by secondary donkey anti-rabbit AlexaFluor594 (1:500; #A-21207, Invitrogen) for 1.5 hours. Fluorescence images were analyzed using a Leica DM5500 fluorescence microscope with OptiGrid structured illumination configuration.

In parallel, another set of sections were incubated with same primary anti-WGA antibody and followed by biotinylated anti-goat secondary antibody (1:1,000; Vector) for 2 hours. Sections were then incubated in the avidin-biotin complex (1:500, ABC; Vector Elite Kit) and incubated in 0.04% 3, 3'-diaminobenzidine and 0.01% hydrogen peroxide. After dehydration through graded ethanol, the slides were then immersed in xylene and coverslipped. Brightfield images were analyzed using a Leica DM5500 microscope.

Validation of DAT-CreER Mice

DAT-CreER/TOMATO mice received i.p. injections of tamoxifen (4 mg/mouse) or vehicle. One week later, mice were perfused with 10% formalin, and brain sections were cut at 25 μm (5 series). The sections were incubated at room temperature in primary rabbit anti-TH antibody

(1:1000, #AB-152, Millipore) overnight, followed by the secondary donkey anti-rabbit Alexa Fluor 488 (1:500; #A-21206, Invitrogen) for 1.5 hours. Co-localization of TOMATO (red) and TH (green) was analyzed using a Leica DM5500 fluorescence microscope with OptiGrid structured illumination configuration.

Training and Assessment of Binge-like Eating Behavior

We used the published protocol (3, 5) to train and assess binge-like eating behavior in mice. Briefly, mice were singly housed and randomly assigned into “intermittent HFD”, “continuous chow/HFD” or “continuous chow only” groups. “Intermittent” mice were initially exposed to both regular chow pellets (6.5% fat, #2920, Harlan) and a high-fat diet (HFD) pellets (40% fat; TD.95217, Harlan) for 48 hours (from Monday 11am to Wednesday 11am), and then exposed to only chow for the rest of the week. Then, weekly cycles were repeated, with both HFD and chow in the cage for 24 hours (from Monday 11am to Tuesday 11am), and only chow for the rest of the week. On the binge assessment day (Mondays of each cycle), HFD and chow intake was measured for 2.5 hours (from 11am to 1:30pm). The “continuous chow/HFD” group was exposed to both chow and HFD for the entire study, and both HFD and chow intake was measured for 2.5 hours at the same time as “intermittent” group. Another control group, “continuous chow only”, was exposed to chow only for the entire study, and chow intake was measured 2.5 hours at the same time as “intermittent” group. The 2.5-hr HFD intake in “intermittent” mice was used to assess binge-like eating behavior in response to various drugs, gene mutations, and their combinations (as detailed below). Mice were housed in their original home cages for the entire training and study period. These cages (W: 7.25 inch, L: 11.5 inch, H: 5 inch; Alternative Design, Cat# RC71U-UD) were made of polysulfone, with a gridded metal

topping holding a water bottle and pellet diets. A metal board was vertically inserted into the food holder to separate chow and HFD pellets.

In order to rule out the possibility that “intermittent” mice binge because of hunger, we exposed a separate cohort wild type mice to intermittent HFD feeding, as described above, to train them to develop binge-like eating. On a binge day, we provided only chow, instead of chow and HFD, and food intake during 11am to 1:30pm was measured. Wild type mice exposed to continuous chow feeding were used as controls. These data were summarized in Figure 1C.

Effects of Fluoxetine and d-Fen on Binge-like Eating Behavior

In experiments in Figure 1D and 1E, male WT and 2C-null littermates (12 weeks of age) were subjected to either “intermittent” HFD exposure or “continuous” chow/HFD exposure, as described above. On the binge assessment days, mice received i.p. injections of saline, fluoxetine (10 mg/kg; Sigma, #F132) or d-Fen (3 mg/kg; Sigma, #F112), at 10:30am (30 min prior to assessment). The same mice received all 3 different injections in different cycles. The order of injections was randomized to avoid potential sequence effects. Note that the doses for fluoxetine was chosen based on a previous report (5) that both 10 mg/kg and 30 mg/kg fluoxetine significantly suppressed binge-like eating in WT mice (5). However, we observed that effects of 30 mg/kg fluoxetine could not be blocked in 2C-null mice (data not shown), suggesting that this high dose of fluoxetine may inhibit binge-like eating through other 5-HT receptors or may have caused toxic effects. Thus, 10 mg/kg was chosen for fluoxetine in the following experiments. Similarly, d-Fen at both 3 mg/kg and 6 mg/kg were tested, and we chose 3 mg/kg because this dose effectively inhibited binge-like eating in WT mice but not in 2C-null mice.

In experiments in Figure 3A, male WT, 2C-null, DA-2C-RE and DA-2C-KO mice littermates (12 weeks of age) received i.p. injections of tamoxifen (4 mg/mouse) and were subjected to “intermittent” HFD exposure. Effects of saline, fluoxetine (10 mg/kg) or d-Fen (3 mg/kg) on binge-like eating behavior (2.5-hr HFD intake) were assessed similarly as described above.

Effects of Lorcaserin on Binge-like Eating Behavior

We first tested effects of lorcaserin on binge-like eating in WT mice. To this end, male C57Bl6 mice (12 weeks of age) were subjected to either “intermittent” HFD exposure or “continuous” chow/HFD exposure, as described above. On the binge assessment days, mice received i.p. injections of saline, lorcaserin (3 mg/kg; MedChem Express, #HY-15368) or lorcaserin (6 mg/kg), at 10:30am (30 min prior to assessment). The same mice received all 3 different injections in different cycles. The order of injections was randomized to avoid potential sequence effects.

After confirm an inhibitory effect of 6 mg/kg lorcaserin, we tested this dose in four mouse groups: WT, 2C-null, DA-2C-RE and DA-2C-KO. Briefly, all mice (12 weeks of age) received i.p. injections of tamoxifen (4 mg/mouse) and were subjected to “intermittent” HFD exposure. Effects of saline, or lorcaserin (6 mg/kg) on binge-like eating behavior were assessed similarly as described above.

In a separate study, WT and DA-2C-KO littermates (before tamoxifen induction) were first subjected to “intermittent” HFD exposure. Binge-like eating behavior were assessed in a cycle before tamoxifen, and assessed again in another cycle after tamoxifen induction (4 mg/mouse). In the following cycles, Effects of saline, or lorcaserin (6 mg/kg) on binge-like

eating behavior were assessed similarly as described above. These data were summarized in Figure S3D and S3E.

Effects of Lorcaserin on Hunger-driven Feeding

Male WT, 2C-null, DA-2C-RE and DA-2C-KO mice littermates (12 weeks of age) received i.p. injections of tamoxifen (4 mg/mouse). These mice were then singly housed and fed with chow only. After an overnight fast, mice received i.p. injections of saline or lorcaserin (6 mg/kg) at 10:30am. Chow diet was provided at 11am, and food intake was measured at 2, 4 and 6 hr time points. The same mice received saline or lorcaserin on separate trials with a one-week interval. The order of injections was randomized to avoid potential sequence effects.

DREADD-induced Activation of DA Neurons and Binge-like Eating Behavior

DAT-CreER mice (male and female, 12 weeks of age) were anesthetized with isoflurane and received stereotaxic injections of the excitatory AAV-hM3Dq-mCherry DREADD virus (200 nl/site; UNC Gene Therapy Center) into both sites of VTA (3 mm posterior, 0.5 mm lateral and 4.5 mm ventral to the Bregma, based on Franklin & Paxinos Mouse Brain Atlas), or into both sites of SN (3.08 mm posterior, 1.25 mm lateral and 4.08 mm ventral to the Bregma, based on Franklin & Paxinos Mouse Brain Atlas). These mice also received i.p. injections of tamoxifen (4 mg/mouse). After a 7-day recovery, mice were subjected to the “intermittent” HFD exposure to induce binge-like eating, as described above. On the binge assessment days, mice received i.p. injections of saline or clozapine N-oxide (CNO, 1 mg/kg; MedChem Express, #HY-17366), at 10:30am (30 min prior to assessment). 2.5-hour HFD intake was measured to assess binge-like

eating. The same mice received both saline and CNO injections in different cycles. The order of injections was randomized to avoid potential sequence effects.

To validate accurate and sufficient infection of AAV-hM3Dq-mCherry in DA neurons within the VTA or the SN, all mice were perfused with 10% formalin 90 min after i.p. injections of saline or CNO (1 mg/kg). Brain sections were cut at 25 μm (5 series). One series were subjected to mCherry immunohistochemistry, and another series were subjected to c-fos immunohistochemistry. Briefly, the sections were incubated at room temperature in primary rabbit DsRed antibody (1:1000, #632496, Clontech), or rabbit anti-c-fos antibody (1:20,000, #PC38, Labome), overnight, followed by biotinylated anti-rabbit secondary antibody (1:1,000; Vector) for 2 hours. Sections were then incubated in the avidin-biotin complex (1:500, ABC; Vector Elite Kit) and incubated in 0.04% 3, 3'-diaminobenzidine and 0.01% hydrogen peroxide. After dehydration through graded ethanol, the slides were then immersed in xylene and coverslipped. Brightfield images were analyzed using a Leica DM5500 microscope. Only those mice with mCherry signals exclusively in the VTA or in the SN were included in analyses for binge-like eating behavior. C-fos immunoreactivity was used to confirm that CNO injections, but not saline injections, activated DA neurons in these mice.

DREADD-induced Inhibition of DA^{VTA} Neurons and d-Fen's Effects on Binge-like Eating Behavior

DAT-CreER mice (male or female, 12 weeks of age) were anesthetized with isoflurane and received stereotaxic injections of the inhibitory AAV-hM4Di-mCherry DREADD virus (200 nl/site; UNC Gene Therapy Center) into both sites of VTA (3 mm posterior, 0.5 mm lateral and 4.5 mm ventral to the Bregma, based on Franklin & Paxinos Mouse Brain Atlas). These mice

also received i.p. injections of tamoxifen (4 mg/mouse). After a 7-day recovery, mice were subjected to the “intermittent” HFD exposure to induce binge-like eating, as described above. On the binge assessment days, mice received pre-injections of saline or CNO (3 mg/kg, i.p.) at 10:00am (60 min prior to assessment), followed by injections of saline or d-Fen (3 mg/kg, i.p.) at 10:30am (30 min prior to assessment). 2.5-hour HFD intake was measured to assess binge-like eating. The same mice received all 4 combinations (2 pre-injections X 2 injections) in different cycles. The order of injections was randomized to avoid potential sequence effects.

To validate accurate and sufficient infection of AAV-hM4Di-mCherry in VTA DA neurons, all mice were perfused with 10% formalin. Brain sections were cut at 25 μm (5 series) and subjected to mCherry immunohistochemistry (by anti-DsRed antibody) as described above. Only those mice with mCherry signals exclusively in the VTA were included in analyses for binge-like eating behavior.

RT-PCR

In order to validate that 5-HT_{2C}Rs are re-expressed selectively in DA neurons in DA-2C-RE mice, WT, 2C-null and DA-2C-KO mice (after tamoxifen induction) were deeply anesthetized with isoflurane and then sacrificed. Various brain regions, including the amygdala, cortex, hypothalamus and VTA, were quickly micro-dissected and stored at -80˚C. As described previously(6), total mRNA was isolated using TRIzol Reagent (Invitrogen) according to the manufacturer's protocol and reverse transcription reactions were performed from 2 µg of total mRNA using a High-Capacity cDNA Reverse Transcription Kits (Invitrogen). Expression of 5-HT_{2C}R was detected in all the tissues with primers that span exon 3 and 4 of 5-HT_{2C}R gene (forward, CTCACTCCTTGTGCACCT; reverse, CCCACCAGCATATCAGCAATG).

Electrophysiology

Mice were deeply anesthetized with isoflurane and transcardially perfused (7) with a modified ice-cold artificial cerebral spinal fluid (aCSF, in mM: 10 NaCl, 25 $NaHCO_3$, 195 Sucrose, 5 Glucose, 2.5 KCl, 1.25 NaH_2PO_4, 2 Na pyruvate, 0.5 $CaCl_2$, 7 $MgCl_2$) (8). The mice were then decapitated, and the entire brain was removed. Brains was quickly sectioned in ice-cold aCSF solution (in mM: 126 NaCl, 2.5 KCl, 1.2 $MgCl_2$, 2.4 $CaCl_2$, 1 NaH_2PO_4, 11.1 Glucose, and 21.4 $NaHCO_3$) (9) saturated with 95% O_2 and 5% CO_2. Coronal sections containing the VTA (250 µm) was cut with a Microm HM 650V vibratome (Thermo Scientific). Then the slices were recovered in the aCSF (9) at 34 ℃ for 1 hr.

Whole-cell patch clamp recordings were performed in the TOMATO-labelled DA neurons in the VTA visually identified by an upright microscope (Eclipse FN-1, Nikon) equipped with IR-DIC optics (Nikon 40x NIR). Signals were processed using Multiclamp 700B amplifier (Axon Instruments), sampled using Digidata 1440A and analyzed offline on a PC with pCLAMP 10.3 (Axon Instruments). The slices were bathed in oxygenated aCSF(9) (32°C–34°C) at a flow rate of approximately 2 ml/min. Patch pipettes with resistances of 3-5 MΩ were filled with solution containing 126 mM K gluconate, 10 mM NaCl, 10 mM EGTA, 1 mM $MgCl_2$, 2 mM Na-ATP and 0.1 mM Mg-GTP (adjusted to pH7.3 with KOH) (10). Current clamp was engaged to test neural firing, resting membrane potential and input resistance. Based on our preliminary data, lorcaserin (30 µM, perfusion upon to 6 minutes at 2 ml/min) were used to examine effects of lorcaserin on DA neurons. In some experiments, the aCSF solution also contained 1 µM tetrodotoxin (TTX) (11) and a cocktail of fast synaptic inhibitors, namely bicuculline (50 µM; a GABA receptor antagonist) (12), AP-5 (30 µM; an NMDA receptor antagonist) (13) and CNQX (30 µM; an AMPA receptor antagonist) (13) to block the majority of

presynaptic inputs. Lucifer yellow dye was included in the pipette solution to trace the recorded neurons. After recordings, slices were fixed with 4% formalin overnight and mounted onto slides. Cells were then visualized with the Leica DM5500 fluorescence microscope to identify post hoc the anatomical location of the recorded neurons in the VTA.

To prove the principle that hM4Dq-infected DA neurons can be inhibited by CNO, a separate cohort of DAT-CreER mice received stereotaxic injections of the inhibitory AAV-hM4Di-mCherry DREADD virus into the VTA as described above. One week after virus infection, mice were sacrificed and VTA-containing brain slices were prepared for electrophysiological recordings in mCherry-labelled DA neurons. Effects of CNO (5 µM, bath superfusion) on resting membrane potential and firing rate were recorded.

Anxiety Tests (Open Field Test, Light-Dark Test and Elevated Plus Maze)

To determine if DA-2C-KO mice have anxiety-related phenotypes, an independent cohort of DA-2C-KO and control littermates were subjected to the open field tests, the light-dark tests, and the elevated plus maze tests, using published protocol (3, 14). Note that all the behavioral tests were performed at around 9-10 am during the light cycles. Briefly, the open-field test was performed in a clear Plexiglas open-field arena (40 cm X 40 cm X30 cm). Mice were first placed into the center of the arena and allowed to explore for 30 min. Overhead lighting and white noise were present to provide ~800 lux illumination and ~55 dB sound inside the arena. Data were collected in 2 min intervals by a computer-operated Digiscan optical animal activity system (RXYZCM, Accuscan Electronics). For the current study, the data over the total 30-min test session were analyzed. Total distance travelled and center time (the time traveled in the center of

the arena) were recorded. Center time can be used to as an index of anxiety-related responses. The number of boli was also measured at the end of testing.

On a different day, these mice were subjected to the light-dark tests. The light-dark test consisted of a polypropylene chamber (44 × 21 × 21 cm) unequally divided into a larger, brightly illuminated open compartment (clear polypropylene) and a smaller, dark compartment (in dark polypropylene), connected by a small opening. Mice were placed in the illuminated chamber and allowed to move freely between the two chambers for 10 min. The latency to enter the light and dark chambers, the time spent in the chambers, the total number of transitions, and the ratio of time in light/dark chamber were measured using the VersaMax Animal Activity Monitoring System (AccuScan Instruments, Inc., OH) and analyzed. Transfer of all four paws of an experimental animal from one chamber to the other was considered as one transition event.

On a different day, these mice were subjected to the elevated plus maze (EPM). The EPM was constructed of plexiglas with two open arms (30 × 5 cm) and two enclosed black arms (30 × 5 × 15 cm) at an elevation of 50 cm above the floor. The arms of the maze form a cross with the two open arms facing each other. The maze was cleaned with 70% ethanol solution after each session and allowed to dry between the sessions. Anxiety-like behavior was measured by placing the mice in the center of the junction of the arms of the maze facing an open arm and the behavior analyzed for 10 min. The numbers of entries into the open and closed arms, the time spent exploring the open and closed arms, and distance traveled were recorded and analyzed using the ANY-maze software (Stoelting Co., Wood Dale, IL). The changes in anxiety-like behavior were calculated by dividing the number of entries into the open arms by dividing the amount of time spent in the open arms by the amount of time spent in all four arms (open time

ratio) and by the total number of entries into all four arms (open entry ratio). The time spent in the center platform not exploring any of the arms was not included in these calculations.

Forced Swim Tests

To determine if DA-2C-KO mice have depression-related phenotypes, DA-2C-KO and control littermates were subjected to the forced swim tests using published protocol (15-17). Briefly, the mice were individually placed into a glass cylinder (25 cm tall X 10 cm diameter) containing 8 cm of water, maintained at 23–25°C for 6 minutes. The mouse movement was digitally recorded from the side using a camcorder. Water in the cylinder was replaced after each recording. The starting and total time of immobility for each mouse was recorded. Immobility is defined as the absence of active, floating with only minimal movements needed to keep head above water. The immobility was scored every 30 seconds for the last 4 minutes. During each 30 seconds, 1 immobility score was recorded if the immobility time was more than 10 seconds. Data from mice that had difficulty keeping heads above water were excluded from analyses. All the videos were blindly rated by a different experimenter.

Physical Activity

Male DA-2C-KO and control littermates were anesthetized by isoflurane and a telemetric Mini Mitter probe ER-4000 (E-mitter; Respironics Inc., Murrysville, PA) was implanted into the abdominal cavity. After a 7-day recovery, mouse cages were put on top of the ER-4000 Receivers (Respironics Inc., Murrysville, PA) (18) to measure physical activity for 5 consecutive days and data were averaged for each mouse.

Statistics

The minimal sample size was pre-determined by the nature of experiments. For most of physiological readouts (food intake, etc.), at least 6 mice per group were included. For histology studies, 2-3 mice were included in each group. For electrophysiological studies, at least 17 neurons in each genotype or condition were included. The data are presented as mean ± SEM. Statistical analyses were performed using GraphPad Prism to evaluate normal distribution and variations within and among groups. Methods of statistical analyses were chosen based on the design of each experiment and are indicated in figure legends. $P<0.05$ was considered to be statistically significant.

Study Approval

Care of all animals and procedures were approved by the Baylor College of Medicine Institutional Animal Care and Use Committee.

Supplemental Results

5-HT_{2C}Rs in DA Neurons Are Required for Full Acquisition of Binge-like Eating in Mice

We noted that the saline-injected 2C-null mice consumed slightly less HFD compared to saline-injected WT mice (Figure S3A). Similar reduction in HFD intake was also observed in saline-injected DA-2C-KO mice, but not in DA-2C-RE mice (Figure S3A). These data suggest that the presence of 5-HT_{2C}Rs in DA neurons is required to train mice to fully acquire binge-like eating behavior, and therefore that 5-HT_{2C}R signals in DA neurons are also involved in the complete development of binge-like eating in mice. To avoid the potential confounding effects of these small differences in saline-treated groups, we analyzed effects of fluoxetine, d-Fen and lorcaserin as relative to saline-treated groups. Consistently, these three drugs significantly suppressed binge-like eating behavior in both WT and DA-2C-RE mice with similar efficacy, but these inhibitory effects were attenuated in 2C-null and DA-2C-KO mice (Figure S3B-S3C).

In order to fully circumvent the confounding effects from differing HFD intake in saline-treated groups, we exposed another cohort of WT and DA-2C-KO mice to intermittent HFD feeding before these mice received tamoxifen induction. Thus, WT and DA-2C-KO mice essentially had the same 5-HT_{2C}Rs expression during the training period, and as expected, these mice showed similar binge-like eating (Figure S3D). Importantly, after tamoxifen induction to delete 5-HT_{2C}Rs from DA neurons in DA-2C-KO mice, the baseline binge-like eating was still comparable between these two groups (Figure S3D). Thus, these results further support the possibility that the endogenous 5-HT_{2C}R signals in DA neurons are required for complete development of binge-like eating. Further, we showed that lorcaserin significantly suppressed binge-like eating in WT mice, but these effects were blunted in DA-2C-KO mice (Figure S3E).

Together, these results indicate that lorcaserin primarily acts through 5-HT_{2C}Rs expressed by DA neurons to suppress binge-like eating behavior in mice.

5-HT_{2C}Rs in DA Neurons Do Not Regulate Physical Activity, Anxiety, and Depression

Since lorcaserin decreases physical activity in rodents (19), we further measured physical activity of WT and DA-2C-KO mice (18), and found no significant difference (Figure S5). Anxiety can also impact feeding behavior and mice lacking 5-HT_{2C}Rs globally are known to have decreased anxiety, although this phenotype has been associated with blunted neural activities in the bed nucleus of the stria terminalis and the central amygdala (20). To rule out the possibility that 5-HT_{2C}Rs in DA neurons may be involved in the regulation of anxiety, we characterized the anxiety levels in both WT and DA-2C-KO mice in various anxiety tests during the light cycle. No significant difference was detected between these two groups in multiple parameters measured in the open field test (Figure S6), in the light and dark field test (Figure S7) or in the elevated plus maze test (Figure S8). We also evaluated the depression levels using the forced swim test, and found no difference between WT and DA-2C-KO mice (Figure S9). Together, these results indicate that 5-HT_{2C}Rs in DA neurons are not required to regulate physical activity, anxiety or depression-like behavior in mice; findings that provide support for the specificity of the function of 5-HT_{2C}Rs in DA neurons in the regulation of binge-like eating behavior.

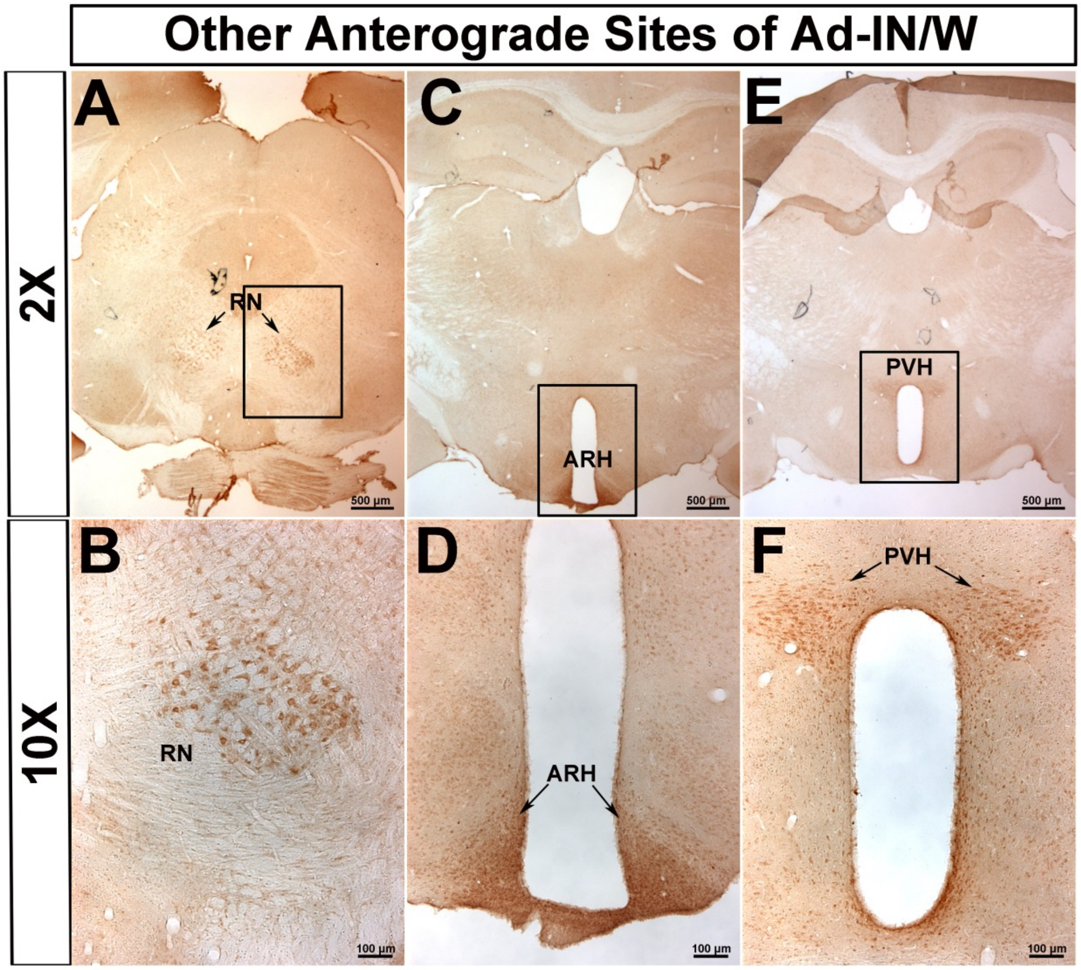

Figure S1. Other anterograde sites of Ad-IN/W injected into the DRN of TPH2-CreER mice. (A, C, E) WGA immunoreactivity in the RN (A), ARH (C) and PVH (E). (B, D, F) are bigger magnification of the black boxes in (A, C, E), respectively. Scales bars are indicated in each image. Arrows in (A-C) point to double-labelled neurons. 3V, 3rd ventricle; ARH, arcuate nucleus of the hypothalamus; RN, red nucleus; PVH, paraventricular nucleus of the hypothalamus.

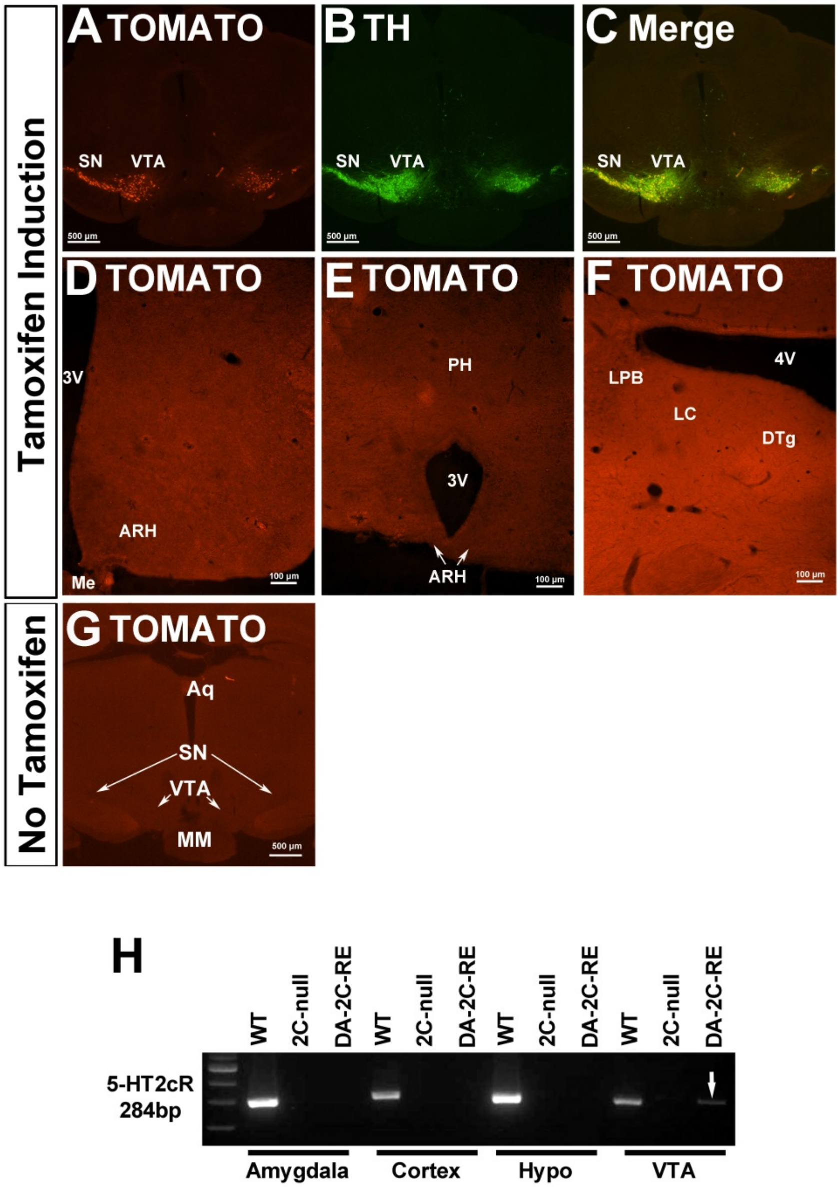

Figure S2. Validation of DAT-CreER mice. (A-F) TOMATO signals in the brain of DAT-CreER/Rosa26-tdTOMATO mice (after tamoxifen induction). TOMATO-labelled neurons were detected in the SN and VTA (A), and co-express TH (B); (C) is the merge of (A) and (B). No TOMATO signals were observed in other brain regions (D-F). (G) The lack of TOMATO signals in the SN and VTA of DAT-CreER/Rosa26-tdTOMATO mice without tamoxifen induction. Scales bars are indicated in each image. 3V, 3rd ventricle; 4V, 4th ventricle; Aq, aqueduct; ARH, arcuate nucleus of the hypothalamus; DTg, dorsal tegmental nucleus; LC, locus coeruleus; LPB, lateral parabrachial nucleus; Me, median eminence; MM, medial mammillary nucleus; PH, posterior hypothalamus; SN, substantia nigra; VTA, ventral tegmental area. (H) RT-PCR detecting 5-HT_{2C}R mRNAs in the amygdala, cortex, hypothalamus (Hypo) and VTA of WT, 2C-null and DA-2C-RE mice. The arrow points to the target band in the VTA of DA-2C-RE mice.

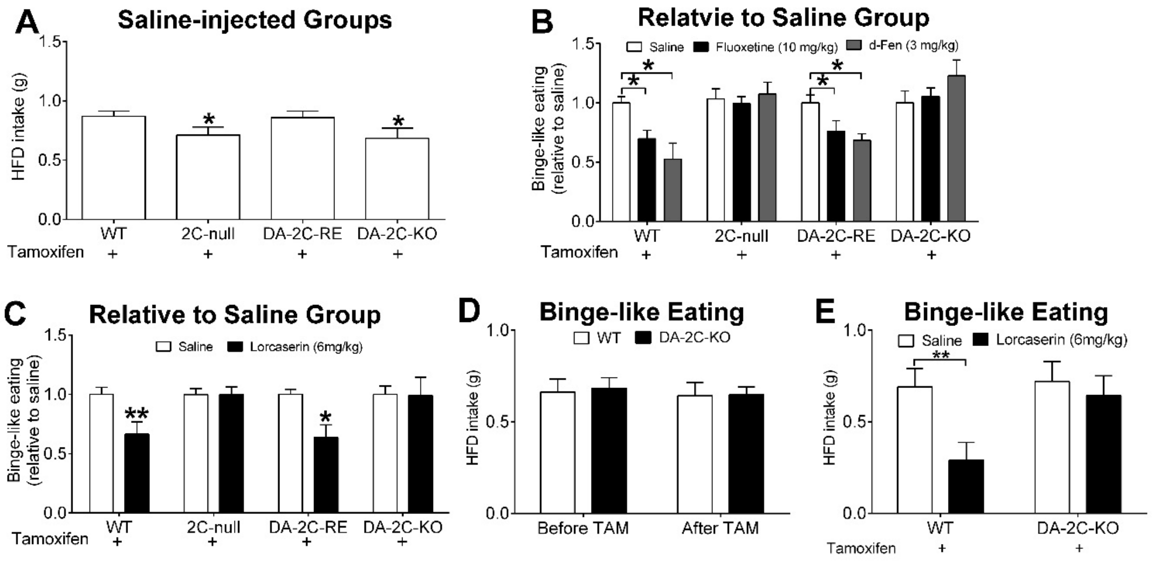

Figure S3. 5-HT$_{2C}$Rs in DA neurons mediate effects of 5-HT drugs to inhibit binge-like eating. (A) Binge-like eating (2.5-hr HFD intake) in saline-injected mice that all received tamoxifen and were exposed to intermittent HFD feeding. Data were retrieved from the saline groups in Figure 3B. N= 6-9/group. Results are shown as mean ± SEM. *, P<0.05 between mutant mice vs. WT mice in one way ANOVA analyses followed by post hoc Sidak tests. (B) Effects of i.p. injections of saline, fluoxetine (10 mg/kg) or d-Fen (3 mg/kg) on binge-like eating (as relative to saline-injected groups) in 4 groups of mice that all received tamoxifen and were exposed to intermittent HFD feeding. Data were retrieved from the saline groups in Figure 3B. N= 6-9/group. Results are shown as mean ± SEM. *, P<0.05 between drug treatment and saline treatment in the same genotype in two way ANOVA analyses followed by post hoc Sidak tests. (C) Effects of i.p. injections of saline or lorcaserin (6 mg/kg) on binge-like eating (as relative to saline-injected groups) in 4 groups of mice that all received tamoxifen and were exposed to intermittent HFD feeding. Data were retrieved from the saline groups in Figure 3D. N= 7-10/group. Results are shown as mean ± SEM. *, P<0.05 and **, P<0.01 between lorcaserin treatment and saline treatment in the same genotype in two way ANOVA analyses followed by post hoc Sidak tests. (D) WT and DA-2C-KO littermates were trained to develop binge-like eating, and then received tamoxifen injections. Binge-like eating (2.5-hr HFD intake) measured on the binge day of 2 repeated cycles, one before tamoxifen injections and one afterwards. N= 5/group. Results are shown as mean ± SEM. (E) Effects of i.p. injections of saline or lorcaserin (6 mg/kg) on binge-like eating (2.5-hr HFD intake) in mice described in (D) in the following cycles. N= 5/group. Results are shown as mean ± SEM. **, P<0.01 between lorcaserin treatment and saline treatment in the same genotype in two way ANOVA analyses followed by post hoc Sidak tests.

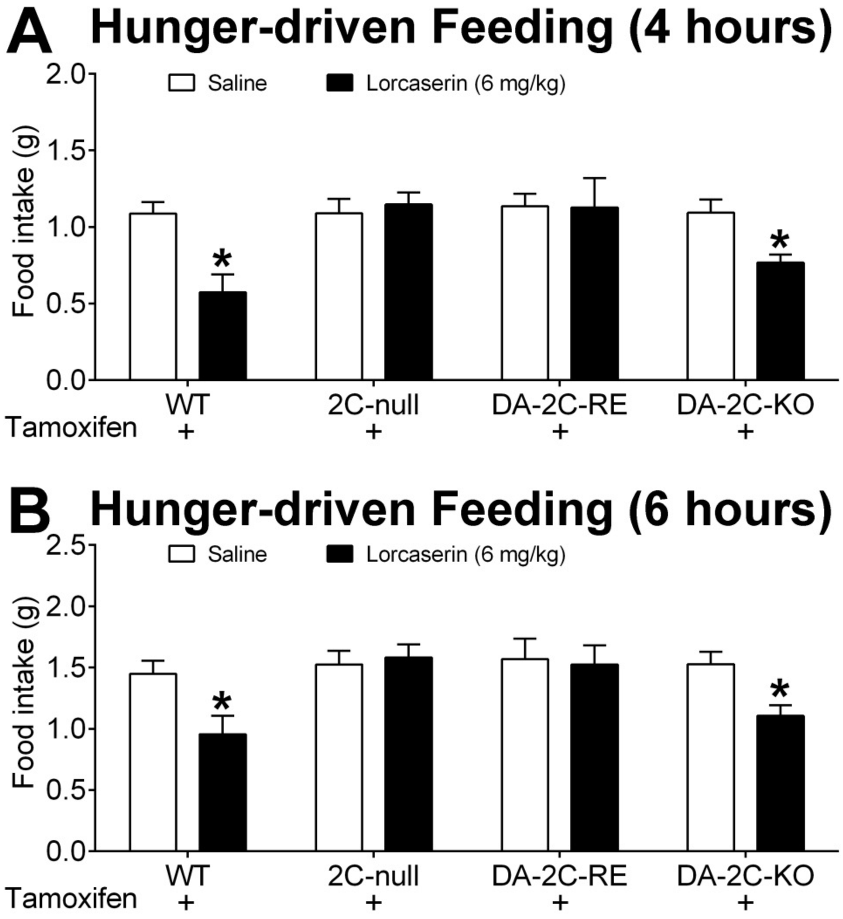

Figure S4. 5-HT$_{2C}$Rs in DA neurons do not mediate effects of lorcaserin on hunger-driven feeding. Effects of i.p. injections of saline or lorcaserin (6 mg/kg) on 4-hr (A) and 6-hr (B) refeeding after an overnight fast in 4 groups of mice that all received tamoxifen and were maintained on chow diet. N= 7-15/group. Results are shown as mean ± SEM. *, $P<0.05$ between lorcaserin treatment and saline treatment in the same genotype in two way ANOVA analyses followed by post hoc Sidak tests.

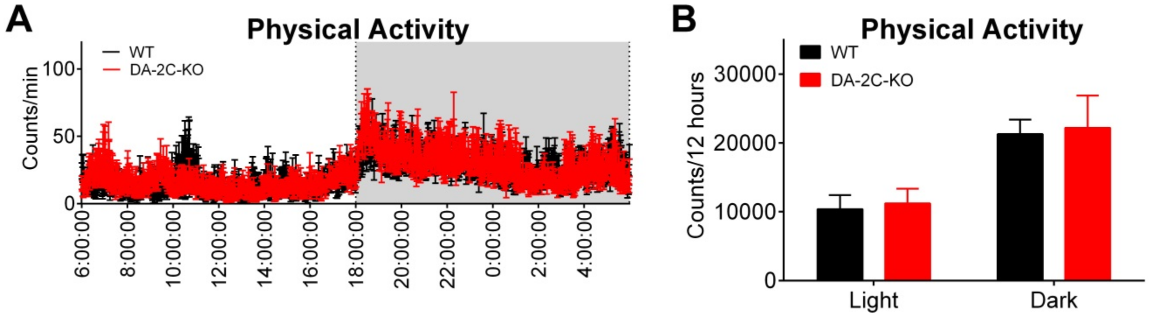

Figure S5. Physical activity. (A) 24-hour physical activity. (B) Sum physical activity during the light or dark cycles. N=4 in each group. Data are presented as mean ± SEM. No significant difference was detected between two groups in t test.

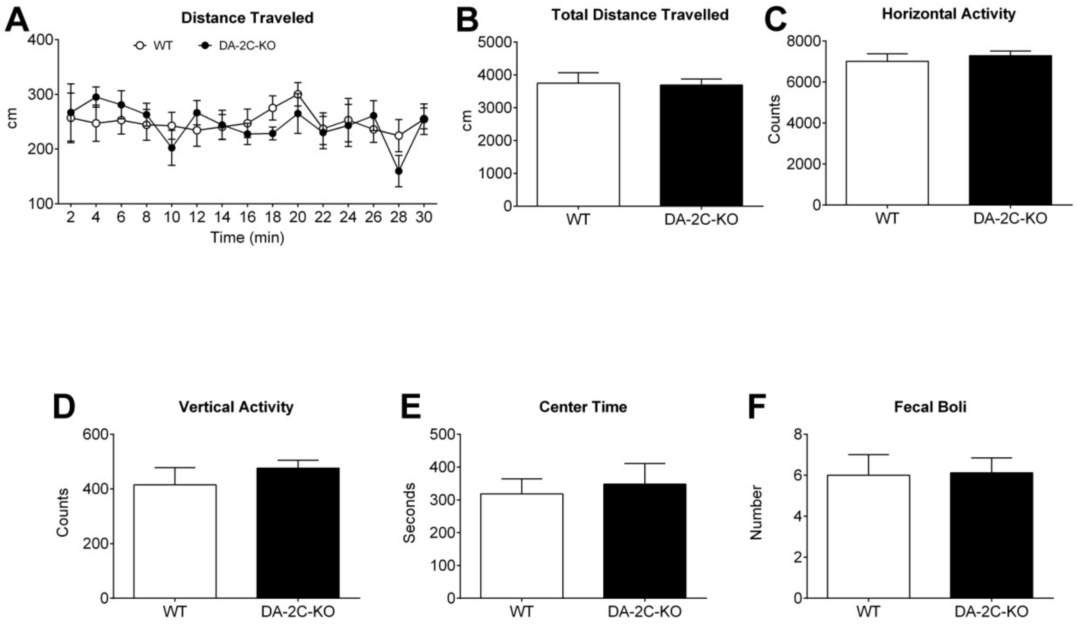

Figure S6. Open field tests. Anxiety-related responses in open field tests in WT and DA-2C-KO mice (8 weeks of age). (A) Distance travelled. (B) Total distance travelled within 30 min. (C) Horizontal activity. (D) Vertical activity. (E) Time spent in the center region of the open field. (F) Numbers of fecal boli. N=8 or 11/group. Results are shown as mean ± SEM. No significant difference was detected between two groups in t-test.

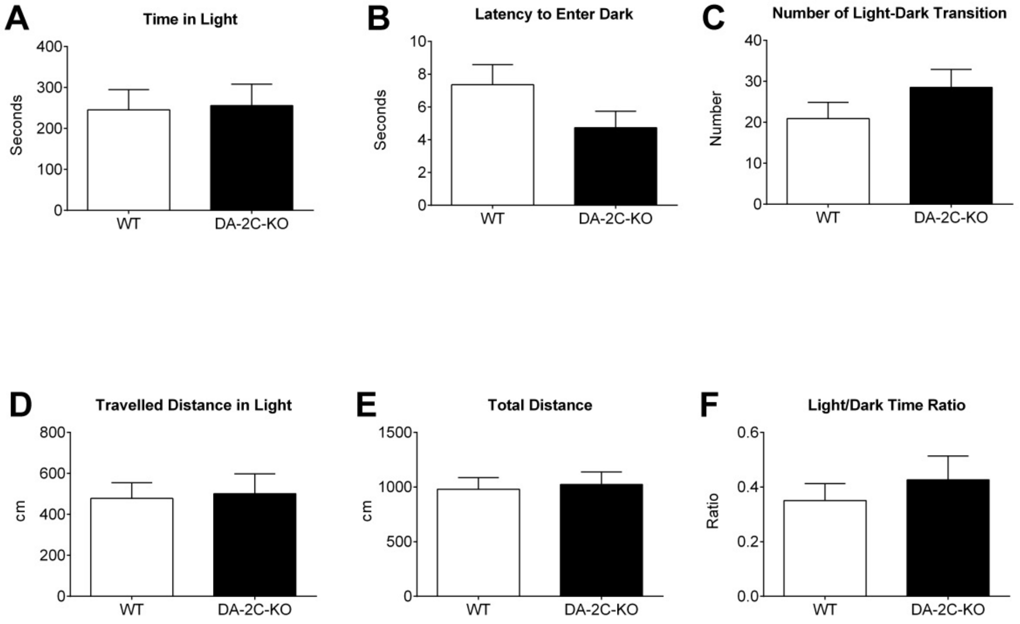

Figure S7. Light-dark tests. Anxiety-related responses in light-dark tests in WT and DA-2C-KO mice (8 weeks of age). (A) Time spent in the light chamber. (B) Latency to enter the dark chamber. (C) The total number of transitions between the light and dark chambers. (D) Distance travelled in the light chamber. (E) Total distance travelled. (F) Ratio of time spent in the light chamber vs the dark chamber. N=8 or 11/group. Results are shown as mean ± SEM. No significant difference was detected between two groups in t-test.

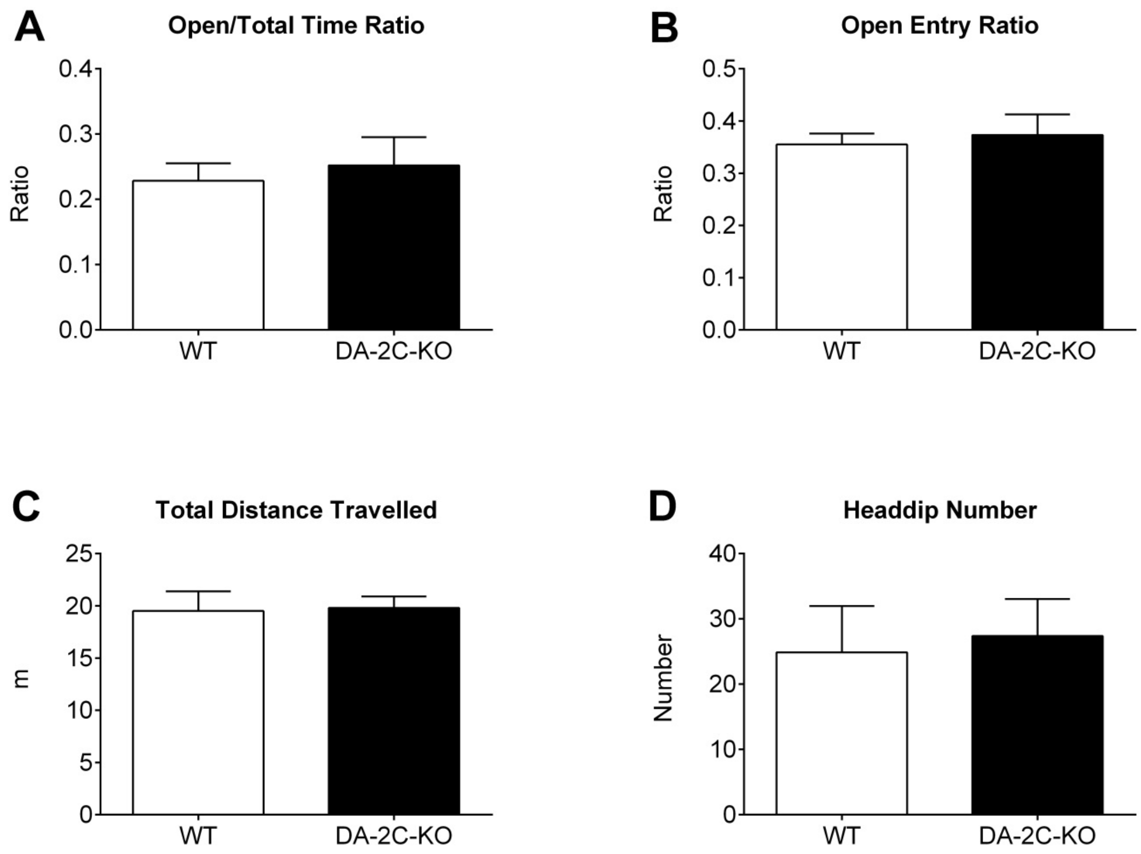

Figure S8. Elevated plus maze tests. Anxiety-like behavior measured in elevated plus maze tests in WT and DA-2C-KO mice (8 weeks of age). (A) Open time ratio. (B) Open entry ratio. (C) Total distance travelled. (D) Numbers of headdip. N=8 or 11/group. Results are shown as mean ± SEM. No significant difference was detected between two groups in t test.

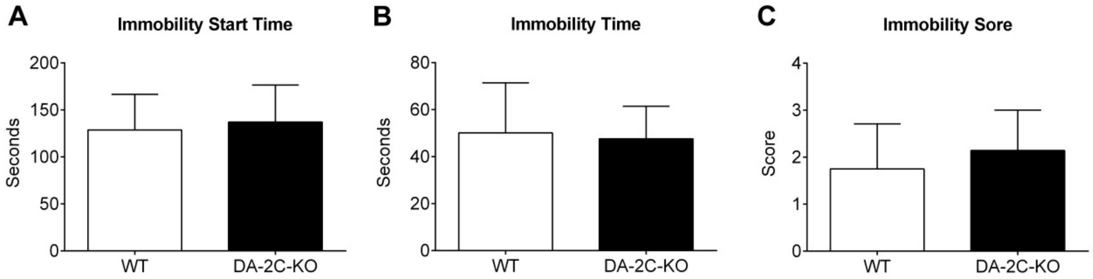

Figure S9. Forced swim tests and physical activity. (A-B) Depression-like behavior measured in forced swim tests in WT and DA-2C-KO mice (8 weeks of age). (A) Immobility start time. (B) Immobility time. (C) Immobility score. N=7 or 8/group. Results are shown as mean ± SEM. No significant difference was detected between two groups in t test.

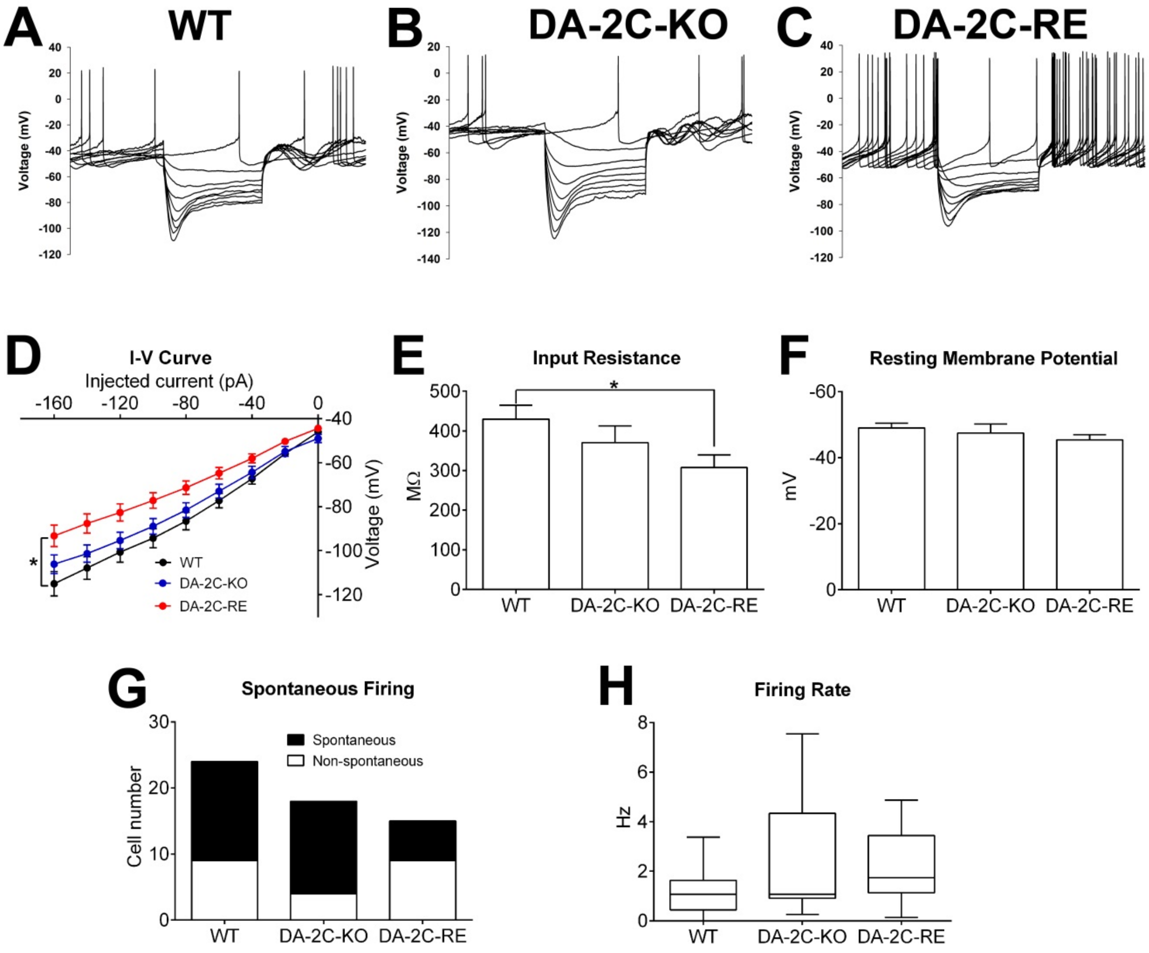

Figure S10. Passive membrane properties of VTA DA neurons from WT, DA-2C-KO or DA-2C-RE mice. (A-C) Representative traces show membrane voltage responses to series depolarizing steps (from -160 pA to 0 pA in steps of 20 pA) for 1 s in VTA DA neurons from WT (A), DA-2C-KO (B) and DA-2C-RE (C) mice. (D) Summary data show current-voltage curves. (E) Input resistance was determined by the slope of a linear regression fitted line of current-voltage curves in (D). N= 9-21/group. Results are shown as mean ± SEM. *, $P<0.05$ in one way ANOVA analyses followed by post hoc Sidak tests. (F) The resting membrane potential in DA neurons. N= 9-21/group. Results are shown as mean ± SEM. (G) Numbers of DA neurons with or without spontaneous firing. (H) The basal firing frequency in the spontaneous firing DA neurons. N= 6-18/group. Results are shown as mean ± SEM.

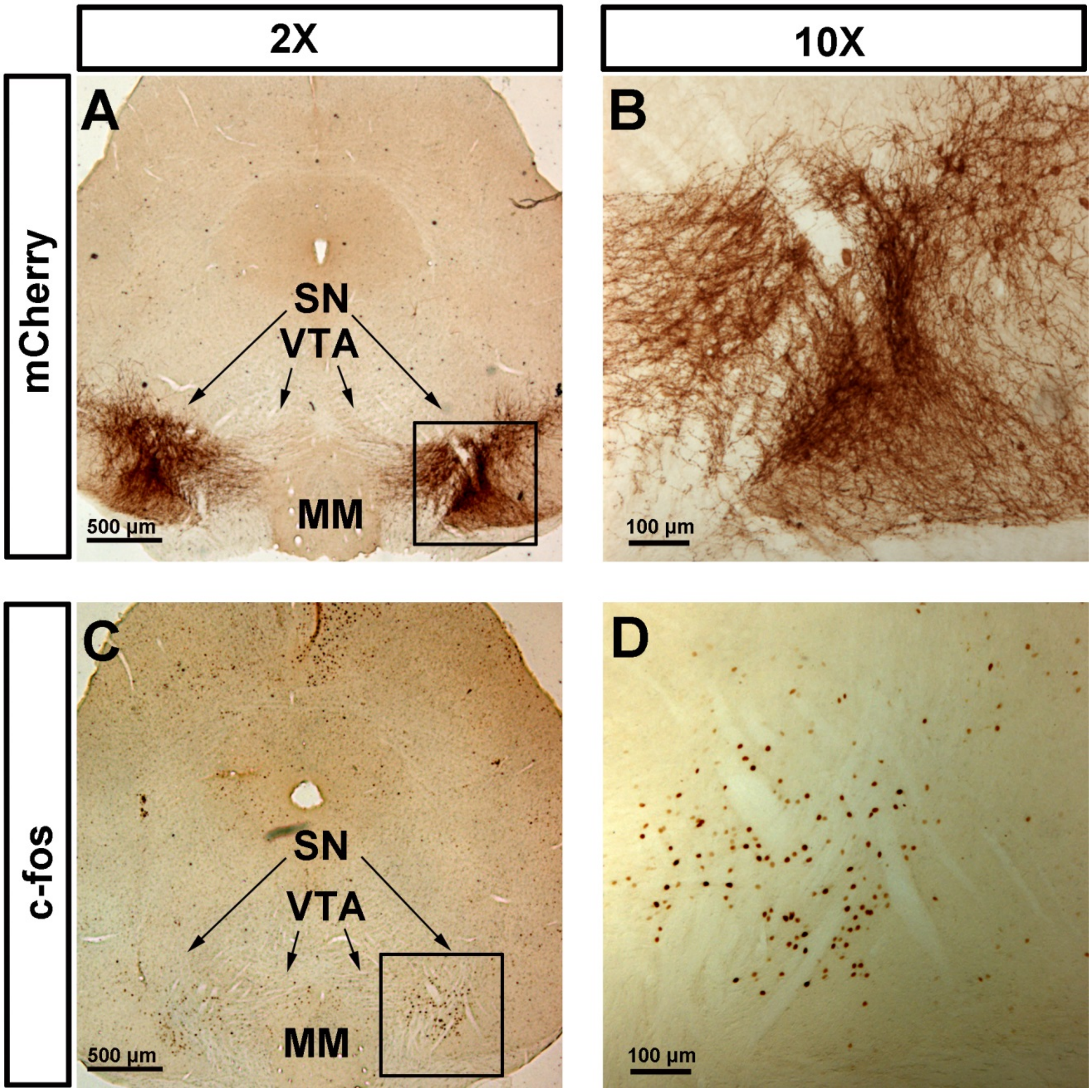

Figure S11. Validation of AAV-hM3Dq-mCherry-mediated activation of DA neurons in the SN. (A-B) mCherry immunoreactivity after injections of excitatory AAV-hM3Dq-mCherry into the SN of DAT-CreER mice. (C-D) c-fos immunoreactivity in the SN 90 min after injections of 1 mg/kg CNO. (B) and (D) are the bigger magnification of the black boxes in (A) and (C), respectively. Scales bars are indicated in each image. MM, medial mammillary nucleus; SN, substantia nigra; VTA, ventral tegmental area.

Supplemental References

1. Xu Y, Jones JE, Kohno D, Williams KW, Lee CE, Choi MJ, et al. (2008): 5-HT2CRs expressed by pro-opiomelanocortin neurons regulate energy homeostasis. *Neuron*. 60:582-589.
2. Berglund ED, Liu C, Sohn JW, Liu T, Kim MH, Lee CE, et al. (2013): Serotonin 2C receptors in pro-opiomelanocortin neurons regulate energy and glucose homeostasis. *J Clin Invest*. 123:5061-5070.
3. Cao X, Xu P, Oyola MG, Xia Y, Yan X, Saito K, et al. (2014): Estrogens stimulate serotonin neurons to inhibit binge-like eating in mice. *J Clin Invest*. 124:4351-4362.
4. Leinninger GM, Opland DM, Jo YH, Faouzi M, Christensen L, Cappellucci LA, et al. (2011): Leptin action via neurotensin neurons controls orexin, the mesolimbic dopamine system and energy balance. *Cell Metab*. 14:313-323.
5. Czyzyk TA, Sahr AE, Statnick MA (2010): A model of binge-like eating behavior in mice that does not require food deprivation or stress. *Obesity (Silver Spring)*. 18:1710-1717.
6. Xu Y, Hill JW, Fukuda M, Gautron L, Sohn JW, Kim KW, et al. (2010): PI3K signaling in the ventromedial hypothalamic nucleus is required for normal energy homeostasis. *Cell Metab*. 12:88-95.
7. Hill JW, Williams KW, Ye C, Luo J, Balthasar N, Coppari R, et al. (2008): Acute effects of leptin require PI3K signaling in hypothalamic proopiomelanocortin neurons in mice. *The Journal of clinical investigation*. 118:1796-1805.
8. Ren H, Orozco IJ, Su Y, Suyama S, Gutierrez-Juarez R, Horvath TL, et al. (2012): FoxO1 Target Gpr17 Activates AgRP Neurons to Regulate Food Intake. *Cell*. 149:1314-1326.
9. Pinto S, Roseberry AG, Liu H, Diano S, Shanabrough M, Cai X, et al. (2004): Rapid rewiring of arcuate nucleus feeding circuits by leptin. *Science*. 304:110-115.
10. Pagadala P, Park CK, Bang S, Xu ZZ, Xie RG, Liu T, et al. (2013): Loss of NR1 subunit of NMDARs in primary sensory neurons leads to hyperexcitability and pain hypersensitivity: involvement of Ca(2+)-activated small conductance potassium channels. *J Neurosci*. 33:13425-13430.
11. Sohn JW, Harris LE, Berglund ED, Liu T, Vong L, Lowell BB, et al. (2013): Melanocortin 4 receptors reciprocally regulate sympathetic and parasympathetic preganglionic neurons. *Cell*. 152:612-619.
12. Liu Y, Liu H, Sauvey C, Yao L, Zarnowska ED, Zhang SC (2013): Directed differentiation of forebrain GABA interneurons from human pluripotent stem cells. *Nat Protoc*. 8:1670-1679.
13. Liu T, Kong D, Shah BP, Ye C, Koda S, Saunders A, et al. (2012): Fasting activation of AgRP neurons requires NMDA receptors and involves spinogenesis and increased excitatory tone. *Neuron*. 73:511-522.
14. Oyola MG, Portillo W, Reyna A, Foradori CD, Kudwa A, Hinds L, et al. (2012): Anxiolytic effects and neuroanatomical targets of estrogen receptor-beta (ERbeta) activation by a selective ERbeta agonist in female mice. *Endocrinology*. 153:837-846.

15. Flaisher-Grinberg S, Einat H (2009): A possible utilization of the mice forced swim test for modeling manic-like increase in vigor and goal-directed behavior. *J Pharmacol Toxicol Methods*. 59:141-145.

16. Pechnick RN, Chesnokova VM, Kariagina A, Price S, Bresee CJ, Poland RE (2004): Reduced immobility in the forced swim test in mice with a targeted deletion of the leukemia inhibitory factor (LIF) gene. *Neuropsychopharmacology*. 29:770-776.

17. Poleszak E, Wlaz P, Kedzierska E, Nieoczym D, Wrobel A, Fidecka S, et al. (2007): NMDA/glutamate mechanism of antidepressant-like action of magnesium in forced swim test in mice. *Pharmacol Biochem Behav*. 88:158-164.

18. Xu P, Cao X, He Y, Zhu L, Yang Y, Saito K, et al. (2015): Estrogen receptor-alpha in medial amygdala neurons regulates body weight. *J Clin Invest*. 125:2861-2876.

19. Higgins GA, Silenieks LB, Rossmann A, Rizos Z, Noble K, Soko AD, et al. (2012): The 5-HT2C receptor agonist lorcaserin reduces nicotine self-administration, discrimination, and reinstatement: relationship to feeding behavior and impulse control. *Neuropsychopharmacology*. 37:1177-1191.

20. Heisler LK, Zhou L, Bajwa P, Hsu J, Tecott LH (2007): Serotonin 5-HT(2C) receptors regulate anxiety-like behavior. *Genes Brain Behav*. 6:491-496.
